# Organic Matter Composition at Ocean Station Papa Affects Its Bioavailability, Bacterioplankton Growth Efficiency and the Responding Taxa

**DOI:** 10.3389/fmars.2020.590273

**Published:** 2020-12-10

**Authors:** Brandon M. Stephens, Keri Opalk, Daniel Petras, Shuting Liu, Jacqueline Comstock, Lihini I. Aluwihare, Dennis A. Hansell, Craig A. Carlson

**Affiliations:** 1 Department of Ecology, Evolution, and Marine Biology, Marine Science Institute, University of California, Santa Barbara, Santa Barbara, CA, United States,; 2 Collaborative Mass Spectrometry Innovation Center, Skaggs School of Pharmacy and Pharmaceutical Sciences, University of California, San Diego, La Jolla, CA, United States,; 3 Scripps Institution of Oceanography, University of California, San Diego, La Jolla, CA, United States,; 4 Department of Ocean Sciences, Rosenstiel School of Marine and Atmospheric Sciences, University of Miami, Miami, FL, United States

**Keywords:** dissolved organic matter, 16S rDNA, Ocean Station Papa, bacterial growth efficiency, total hydrolyzable amino acids, LC-MS/MS, organic matter remineralization, alpha diversity

## Abstract

The bioavailability of organic matter (OM) to marine heterotrophic bacterioplankton is determined by both the chemical composition of OM and the microbial community composition. In the current study, changes in OM bioavailability were identified at Ocean Station Papa as part of the 2018 Export Processes in the Ocean from Remote Sensing (EXPORTS) field study. Removal rates of carbon (C) in controlled experiments were significantly correlated with the initial composition of total hydrolyzable amino acids, and C removal rates were high when the amino acid degradation index suggested a more labile composition. Carbon remineralization rates averaged 0.19 ± 0.08 μmol C L^−1^ d^−1^ over 6–10 days while bacterial growth efficiencies averaged 31 ± 7%. Amino acid composition and tandem mass spectrometry analysis of compound classes also revealed transformations to a more degraded OM composition during experiments. There was a log2-fold increase in the relative abundances of 16S rDNA-resolved bacterioplankton taxa in most experiments by members of the *Methylophilaceae* family (OM43 genus) and KI89A order. Additionally, when OM was more bioavailable, relative abundances increased by at least threefold for the classes *Bacteroidetes* (*Flavobacteriaceae* NS2b genus), *Alphaproteobacteria* (*Rhodobacteraceae Sulfitobacter* genus), and *Gammaproteobacteria* (*Alteromonadales* and *Ectothiorhodospiraceae* orders). Our data suggest that a diverse group of bacterioplankton was responsible for removing organic carbon and altering the OM composition to a more degraded state. Elevated community diversity, as inferred from the Shannon-Wiener *H* index, may have contributed to relatively high growth efficiencies by the bacterioplankton. The data presented here shed light on the interconnections between OM bioavailability and key bacterioplankton taxa for the degradation of marine OM.

## INTRODUCTION

Ocean Station Papa (OSP), located in the subarctic NE Pacific, experiences seasonal cycles in bacterioplankton biomass and productivity (Kirchman et al., 1993; Boyd et al., 1995; Sherry et al., 1999). Both net primary and bacterioplankton production (BP) nearly double at OSP in spring and summer relative to winter (Sherry et al., 1999) despite iron limitation that leads to high-nutrient low-chlorophyll conditions (Martin and Fitzwater, 1988; Boyd and Harrison, 1999; Harrison et al., 1999). BP can represent up to ~25% of primary production (Sherry et al., 1999), exhibiting the greatest partitioning of primary production into BP in summer months. Such rate comparisons demonstrate that bacterioplankton can comprise a sizable portion of the carbon (C) demand at OSP, though there still remains uncertainty as to the contributions of top-down (predation) vs. bottom-up (organic matter supply) controls on BP (Kirchman et al., 1993; Doherty, 1995; Sherry et al., 1999).

Bacterioplankton are limited to utilizing low molecular weight (LMW) (<600 Da; Weiss et al., 1991) dissolved organic matter (DOM) and so must hydrolyze high molecular weight or particulate organic matter to LMW compounds to consume it (Amon and Benner, 1994; Arnosti et al., 2005; Arnosti, 2011). At OSP, short-term radiotracer-based experiments (conducted over hours) demonstrated that bacteria were primarily limited by the supply of DOM, particularly as dissolved amino acids (Kirchman et al., 1989, 1993; Kirchman, 1990; Sherry et al., 1999). Bacteria found in other domains also exhibit enhanced growth and growth efficiencies when grown on amino acids compared with other substrates like sugars (Russell and Cook, 1995), illustrating the importance of this LMW DOM substrate for bacterioplankton growth. Although bacterial dynamics were established decades prior, the seasonal dynamics of dissolved organic carbon (DOC) concentrations at OSP were only recently put into the global context (Bif and Hansell, 2019; Lopez et al., 2020). DOC concentrations exhibit elevated concentrations in surface waters during summer months then decrease in winter, providing further evidence of the link between the supply of DOM and elevated bacterial growth in summer at OSP.

While seasonal heterotrophic bacterial production data based on ^3^H-Leucine incorporation rates at OSP have identified a link between primary and bacteria production (Kirchman et al., 1993; Sherry et al., 1999), long-term (spanning days to weeks to months) DOM remineralization experiments have not yet been conducted in the NE Pacific. The original design for such experiments consisted of inoculating a 0.6 μm pre-filtered natural assemblage of bacteria into 0.2 μm-filtered water at a 50–70% dilution to reduce the effects of bacterivory and to assess bacterial community changes solely due to growth on DOM (Ammerman et al., 1984). These experiments have since been modified and conducted over timescales of days to weeks to months to quantify the fate of accumulated DOM (Carlson and Hansell, 2015). In addition to quantifying the removal rates of DOM (i.e., DOM bioavailability) and the growth of bacterioplankton to derive bacterial growth efficiencies (BGEs) (Carlson and Ducklow, 1996), these experiments can be used as platforms to assess the transformation of DOM composition as well as concomitant shifts in bacterioplankton community structure (Wear et al., 2015; Liu et al., 2020).

The current study presents data from OM remineralization experiments in order to (1) estimate the rates of organic C removal; (2) characterize the initial OM composition and subsequent changes associated with OM removal; (3) estimate bacterial growth dynamics and associated growth efficiencies for various depths; and (4) identify bacterial community composition changes associated with varying OM consumption. The experiments presented here found that, surprisingly, despite a stable water column, the rates of C remineralization were variable over the 3-week cruise period and across depths. Overall, surface water OM bioavailability increased as the initial composition of the OM was more labile, as inferred from the composition of amino acids. High OM bioavailability was associated with increased relative abundances of specific bacterioplankton taxa. Shifts toward a more diverse bacterioplankton community were observed in most experiments, and greater increases in diversity were associated with a more degraded OM signature with incubation time. Data also highlight specific bacterial taxa and community diversity associated with the utilization of accumulated C at OSP.

## MATERIALS AND METHODS

### Study Region

This study was conducted as part of the Export Processes in the Ocean from Remote Sensing (EXPORTS) program aboard the R/V *Roger Revelle* cruise RR1813 (operating in a Lagrangian framework) near OSP (50.1°N, 144.9°W) between August 15 and September 7, 2018. A Lagrangian float tracked a coherent mesoscale feature over three 8-day intervals. At the time of sampling, the surface waters had a mixed layer depth of ~30 m and temperatures were ~14°C between 5 and 30 m (Supplementary Figure 1), decreasing to ~8.5°C at 50 m and ~6°C at 95 m (McNair and Menden-Deuer, 2020). The mean 1 and 0.1% photosynthetically active radiation depths were 78 ± 6 m and ~110 m, respectively (McNair and Menden-Deuer, 2020). Data for the current study can be found at http://dx.doi.org/10.5067/SeaBASS/EXPORTS/DATA001 (Werdell et al., 2003).

### Remineralization Experiments

#### Experimental Setup

Seawater for the experiments was collected from 5, 35, 50, and 95 m depths. Water was gravity-filtered from Niskin bottles through in-line 142 mm pre-flushed 0.2 (Millipore Sigma GSWP14250) or 3.0 μm (Millipore Sigma SSWP14250) mixed cellulose ester filters into acid-washed polycarbonate carboys after rinsing with seawater 3 times. Incubation types included: (1) 3.0 μm filtrate “*undiluted*” experiments, and (2) “*diluted*” experiments, in which 3.0 μm-filtered inoculum was diluted by 70% with 0.2 μm filtrate collected from the same depth (i.e., 70% < 0.2 μm: 30% < 3.0 μm). As such, the bulk organic carbon measured in all samples collected from *diluted* and *undiluted* experiments presented in this study was at least < 3.0 μm.

The high temperature combustion method (see below) and associated error was not able to differentiate between the organic carbon concentrations measured in the <0.2 μm and <3.0 μm filtrates (Supplementary Figure 2), though we recognize the potential contribution of colloidal and gel-like organic materials between 0.2 and 3.0 μm in addition to bacterial carbon. To minimize handling error, samples for OM were not re-filtered at each time point, but rather we subtracted the contribution of bacterial carbon. Thus, the measured organic carbon concentration corrected for bacterial biomass is termed “DOC*”.

After filtration and mixing, water for the experiments was gently poured into replicate 5 L polycarbonate bottles (‘Biotainer,’ ThermoFisher) and incubated in the dark at the *in situ* temperature (± 3°C) of inoculum source water (14°C for 5 and 35 m, and 6°C for 50 and 95 m samples) in an Isotemp incubator (Thermofisher, model 3720A). The caps of the 5 L Biotainers were outfitted with bulkhead fittings that allowed the headspace to be pressurized at the time of sampling (~3.4 psi) by air from an aquarium pump (Fluval Q2, model A852) after passing a charcoal (Restek, model 22013) and air filter (Pall Vacushield, model 4402). The applied pressure positively displaced water from the carboy through a Teflon tube that extended from the bottom of the Biotainers, then through the cap to a sampling tube with an open Luer lock fitting. In-line filter cartridges were attached to the Luer lock fitting when further sample filtration was necessary; see Liu et al. (2020) for results from a similar experimental design.

After filling the 5 L Biotainers with experimental water, a subset of the mixed experimental water was displaced into 24 pre-combusted (450°C) 40 mL borosilicate replicate vials (sample-rinsed 3 times ea.) that served as parallel incubations (referred to as “parallel vials”); these were placed next to the Biotainers in the same incubator. The parallel vial incubations were conducted in order to (1) extend the monitoring of experiments beyond the cruise duration; (2) to minimize handling for DOC* samples; (3) minimize use of waters in the Biotainers, thus minimizing changes in their surface area:volume ratio due to the required removal from those of liters of sample for DNA and bacterial biomass sampling at the T0 and onset of stationary growth phase. Triplicate samples for DOC* and single samples for bacterial biomass (BB) were collected from the 5 L Biotainers and 3 replicate sacrificed parallel incubation vials at different time intervals during incubations (Table 1). The measured bacterioplankton response in the parallel vials agreed to within 5% of that observed in the 5 L Biotainers, indicating little difference due to unequal experiment volumes (Supplementary Figure 3).

Growth curves, based on changes in bacterioplankton abundance (see below), were generated for each experiment. Stationary growth phase was identified as that period demonstrating no cell growth with time. At stationary phase, water was collected (see below) for the extraction of DNA and DOM. The 5 L Biotainer experiments were terminated at stationary phase while still aboard the research vessel; the parallel vial incubations, continued past stationary phase, were shipped to the University of California, Santa Barbara (UCSB) overnight following the cruise, placed in incubators and monitored for up to ~90 days after initiation of the experiment.

#### Bacterioplankton Abundances and Sizes

Change in bacterioplankton abundance in the experiments was monitored daily using a shipboard flow cytometer (Guava, Millipore). Samples were fixed with 1% (final concentration) paraformaldehyde, stained with SYBR Green I dye (ThermoFisher) and quantified daily following procedures detailed in Gasol and Morán (2015). Post-cruise cell abundance samples were validated with epifluorescence microscopy and image analysis at UCSB. Samples for microscopy were collected into sterile centrifuge tubes (Corning) and fixed with 0.2 μm filtered formalin at 1% of the final volume in the sample. The samples were stained with 5 μg mL^−1^ 4′,6-diamidino-2-phenylindole dihydrochloride (DAPI, Sigma-Aldrich; Porter and Feig, 1980), then enumerated and sized via epifluorescence microscopy using a Revolve microscope (Discover Echo Inc.) with a 60x objective and ImageJ image analysis software. ImageJ code can be accessed at https://seabass.gsfc.nasa.gov/archive/UCSB/carlson/EXPORTS/EXPORTSNP/documents.

Briefly, cells were identified and sized using Gaussian blur (sigma of 10) background suppression, contrast enhancement (~15%) and an Otsu-based thresholding algorithm (Otsu, 1979). Cell sizes were calibrated using standard fluorescent beads (Thermofisher; sized 0.1, 0.2, 0.5, 1.0, and 4.0 μm in diameter); maximum and minimum cell dimensions determined cell biovolumes. Mean cell biovolumes for each image were estimated using an R script that calculated biovolumes based either on an assumed spherical diameter:

(1) $$\text{Spherical}-\text{shaped}\;\text{Cell}\;\text{Biovolume}=4/3\left(\pi r^{3}\right)$$

when the ratio of maximum to minimum dimensions was <1.5, or on an assumed rod:

(2) $$\text{Rod}-\text{shaped}\;\text{Cell}\;\text{Biovolume}=4/3\left(\pi r^{3}\right)+\pi r^{2}h$$

where *r* = radius of the cell and *h* = maximum dimension - minimum dimension (Baldwin and Bankston, 1988). This method cannot differentiate between bacteria and archaea; thus, the combined groups are referred to here as bacterioplankton, with abundances converted to carbon biomass as detailed below.

#### Bacterioplankton Biomass (BB)

A 1 L water sample, collected at the initial and stationary-growth phases, was concentrated on pre-combusted GF/75 filters (0.3 μm nominal pore-size and 25 mm diameter, Cole Parmer) double-stacked within an acid washed 25 mm polypropylene filter cartridge. Filters were saved in individual pre-combusted glass vials for elemental carbon and nitrogen quantification at Bigelow Laboratories for Ocean Sciences using a Costech ECS 4010 elemental analyzer (980°C combustion temperature) (James et al., 2017). Both top and bottom filters were used to estimate cell carbon. This mass was blank-corrected by passing 30 kDa tangential filtrate through double-stacked GF/75 filters. The particle free 30 kDa filtrate represented DOM sorption to the active sites on the GF/75 filters and was an average of 5.3 ± 1.3 μg C L^−1^. The GF/75 blank was similar to the carbon collected on the bottom GF/75 filters after filtering experiment samples (5.1 ± 1.7 μg C L^−1^) and remained relatively constant despite varying initial DOC concentration from different depths. Cell abundances of unfiltered water and GF/75 filtrate showed that an average of 78.3 ± 9% cells was retained by GF/75 for initial and stationary growth phases. BB was determined using the cell carbon relationship such that carbon per cell (fg C cell^−1^) = 91.71* (cell biovolume in μm^3^)^0.686^ (Supplementary Figure 4).

#### Organic Carbon

Three borosilicate vials were sacrificed and fixed per time point by adding 50 μl DOC-free 4N HCl to 35 ml samples (final pH < 3). Upon returning to UCSB, samples were stored at ~14°C in an environmental chamber free of volatile organics until analysis. Organic carbon concentrations were analyzed on modified Shimadzu TOC-V or TOC-L analyzers following Carlson et al. (2010). Concentrations were quantified using glucose standard solutions with UV-irradiated Nanopure (low carbon) water. All samples were systematically referenced against surface (5 m) and deep (3000 m) Pacific seawater that were calibrated against consensus reference material (Hansell SSR Lot#08-18) and run every 6 – 8 samples and blank corrected with values derived from UV-irradiated Nanopure water (Hansell and Carlson, 1998). Typical run sizes were kept under 35 samples to reduce salt accumulation and instrument drift. The precision of the Shimadzu analyzers for surface samples was within 0.7 μM C on average for the EXPORTS dataset reported here. Bacterioplankton biomass (above) was subtracted from each time point’s measure of organic carbon to derive DOC*.

#### Bacterial Growth Efficiency (BGE)

Coupling changes in bacterioplankton biomass production rates with statistically significant short-term DOC* removal rates (i.e., 6–10 days) allowed us to constrain BGE values in *diluted* and *undiluted* experiments. BGE was estimated, similar in form to Carlson and Ducklow (1996), using the following formulation:

(3) $$\text{BGE}=\text{BP}/\text{DO}C^{*}\text{removal}\;\text{rate}$$

where BP represents the net BP rate determine from the model I linear regression of BB vs. time from T0 to stationary phase and DOC* removal rate is determined by the model I linear regression of DOC* vs. time from T0 to stationary phase. We only report BGEs when the change in DOC* between T0 and stationary phase exceeded twice the mean instrumental uncertainty (2x of 0.7 μmol C L^−1^ = 1.4 μmol C L^−1^) and when the combined linear regression models of BP and DOC* removal rate were statistically significant (two-tailed *t*-tests *p* < 0.05).

#### Total Hydrolyzable Amino Acids

Samples for total hydrolyzable amino acid (THAA) analysis were taken from acidified parallel vials. Preliminary tests showed no significant difference in THAA mol% composition between samples stored frozen vs. stored at 14°C at a pH of ~3. THAA analysis was modified from a combination of previously published studies (Lindroth and Mopper, 1979; Henrichs, 1991; Cowie and Hedges, 1994; Kaiser and Benner, 2009; Liu et al., 2020). Samples, UV-irradiated Nanopure blanks (Thermo Scientific), and Sargasso Sea reference water (1 m water collected in 2018 and stored frozen) were sealed in ampoules under nitrogen and hydrolyzed using 6N HCl (Optima grade) at 110°C for 20 h. Hydrolyzed samples were neutralized via evaporation and detected by a Dionex RF2000 Fluorescence Detector (Ex = 330 nm, Em = 418 nm) after automated addition of *o*-phthalaldehyde (OPA) within a Dionex autosampler at 10°C.

A Dionex Acclaim 120, C18 (5 μm, 120 A, 4.6 × 250 mm) column, with a guard column, separated amino acids (AAs) using a gradient modified from prior studies highlighted above. Briefly, the gradient began with 77% sodium acetate (50 mmol L^−1^, pH 5.7) and 23% methanol, then shifted to 29% methanol at 4 min, 44% methanol at 20 min, 60% methanol at 33 min, 77% methanol at 48 min and 100% methanol at 53 min. Eighteen amino acids were detected, with integrated peaks calibrated using a set of standards at four concentrations (5–250 nmol L^−1^ for each AA). THAA concentrations were used to calculate the degradation index (DI) score (Dauwe et al., 1999; Kaiser and Benner, 2009; Liu et al., 2020) and the combined mol% of the non-protein amino acids gamma-aminobutyric acid (GABA) and beta-alanine (B-Ala) (Cowie and Hedges, 1994; Dauwe and Middelburg, 1998; Amon et al., 2001).

#### PPL Solid-Phase Extraction and LC-MS/MS Analysis of DOM

Dissolved organic matter from 1 L of 0.2 μm Sterivex-filtrate (Sterivex filter used for DNA collection as noted below), collected at initial and stationary phases, was acidified to ~pH 2 (ACS grade HCl) for isolation via solid-phase extraction using Priority PolLutant (PPL) cartridges (1 g Bond Elut, Agilent) according to Petras et al. (2017). The cartridges were prepared by adding 3 bed volumes of 100% methanol (LC-MS grade) and the residual methanol displaced by nitrogen gas. Samples were then passed through the cartridges at ~13 mL min^−1^. Residual seawater was similarly pushed out of the cartridges and the cartridge stored at −80°C until further processing at UCSB. The PPL cartridges that contained sample were completely dried using high purity grade nitrogen. DOM was then eluted with an addition of 2 bed volumes (~6 mL) of 100% methanol (LC-MS grade). Extracts were dried using high purity grade nitrogen and resuspended in 6 mL of LC-MS grade methanol.

Analysis of PPL-extracted DOM was performed by liquid chromatography-tandem mass spectrometry (LC-MS/MS) with an ultra-high-performance liquid chromatograph (UHPLC) coupled to a Q-Exactive orbitrap mass spectrometer (Thermo Fisher Scientific, Bremen, Germany) following Petras et al. (2017). The relative abundances of MS1 features > 3x process blank peak heights were determined for each sample and converted to *z*-scores (see below). Process blanks were generated in a similar manner as for samples; 1 L of LC-MS grade water was first filtered through a Sterivex cartridge then acidified to pH 2 and finally passed through the PPL cartridge.

*Z*-scores for each molecular feature were determined by taking the relative abundance of a feature in a sample minus average relative abundance of that feature across all samples and then dividing by the standard deviation of that feature across all samples. A decrease in LC-MS/MS peak area *z*-scores indicated a decrease in the abundance of those compounds with time. The spectra were submitted to Ion-Identity Molecular Networking (Schmid et al., 2020) in Global Natural Product Social Molecular Networking (GNPS) site to create a molecular network and were then searched against GNPS spectral libraries and National Institute of Standards and Technology Library 17. The approach described here considers the annotated features to be ‘putative’ identifications that have not yet been verified by reference standards, but are based on spectral similarity to data from public or commercial libraries (Sumner et al., 2007; Longnecker et al., 2015; Longnecker and Kujawinski, 2017). Using library and analog matches, we categorized the molecular features within molecular networks (Aron et al., 2020) into six broad compound classes. Compound classes are based on International Chemical Identifiers of known library and analog matches in Classyfire (Djoumbou-Feunang et al., 2016). Further LC-MS/MS methods details can be found in Supplementary Text 1.

#### 16S rDNA Amplicon Sequencing

At initial and stationary phases, 1 L of sample was concentrated on 0.2 μm polyethersulfone filter cartridges (Sterivex-GP, Millipore) then stored at −80°C according to Liu et al. (2020). One mL of sucrose lysis buffer (40 mmol L^−1^ EDTA, 50 mmol L^−1^ Tris-HCl, 750 mmol L^−1^ sucrose, 400 mmol L^−1^ NaCl, pH adjusted to 8) was immediately added to the filters after filtration. DNA was extracted from filters using phenol:isoamyl alcohol:chloroform (PIC, in 25:1:24 ratios) following Giovannoni et al. (1996). Extracted DNA concentrations were quantified on a Qubit 4 Fluorometer (ThermoFisher Scientific) after resuspending pellets in 20 μL of polymerase chain reactions (PCR)-grade water. DNA concentrations across all depths ranged 2–230 ng μL^−1^ (mean 49 ± 42), compared with a PCR water process blank of 0.1 ng μL^−1^.

The 16S rRNA gene was amplified in 25 μl PCR reactions using the V4 primers (515F-Y and 806RB, Apprill et al., 2015; Parada et al., 2016) and a Bio-Rad Tetrad 2 thermal cycler following the Robust HotStart ReadyMix protocols (KAPA, Roche). PCR reactions were cycled for 3 min at 95°C; 30 cycles of 30 s at 95°C, 30 s at 57°C, and 1 min at 72°C; and 10 min at 72°C. Reactions were cycled in PCR-grade blank water and two mock communities were included with each 96-well plate of samples as quality control checks (BEI Resources mock communities HM-782D and HM-783D and a custom mock community from the Santa Barbara Channel; Wear et al., 2018). Amplified samples were sequenced on an Illumina MiSeq and demultiplexed at UC Davis’ Genome Center.

Amplicon sequencing reads were trimmed and assigned to taxonomies based on a DADA2 pipeline (Callahan et al., 2016) using matches to the SILVA SSU/LSU 132 database (accessed in December of 2019). After plastid sequences (e.g., chloroplasts and mitochondrial) were removed, samples had read depths ranging 10,951–44,686 reads (average of 18,940 ± 7,010) and resulted in 492 unique amplicon sequence variants (ASVs) across the 21 16S rDNA samples. A phylogenetic tree was created using the RAxML (v8.2.10) program (Stamatakis, 2014) running 100 bootstraps on a nucleotide GTRGAMMA model of rate heterogeneity. For later use in generating UniFrac distance matrices (Lozupone and Knight, 2005), the phylogenetic tree associated with non-rarefied relative abundances was used to create a ‘phyloseq’ (v1.32.0) object (McMurdie and Holmes, 2013, 2014). The phyloseq object was then used in non-metric multidimensional scaling (nMDS) ordination analysis based on weighted UniFrac distances and was used in estimating alpha diversity metrics.

### Statistical Analyses

All statistical tests were considered significant at the *p* < 0.05 level, and all results are ± standard deviations, unless otherwise stated. The Shapiro-Wilk test (Shapiro and Wilk, 1965) determined if data were normally distributed, indicating whether to use non-parametric-based statistics to evaluate for significance within or between datasets. For normal distributions, a two-sample *t*-test was used and for non-normal distributions a Mann-Whitney test (Mann and Whitney, 1947) was used to compare whether given samples were statistically different. As noted above, BP and DOC* removal rate were best fit with linear regression models and the significance of BGEs determined using the combined model errors. The combined error for the BGE values was estimated using the following formulation:

(4) $$\text{BGE}\;\text{Error}=[{\left(\text{DO}C^{*}\;\text{removal}\;\text{rate}/\text{DOC}{\;}^{*}\;\text{removal}\;\text{rate}\;\text{error}\right)}^{2}{+{\left(\text{BP}/\text{BP}\;\text{error}\right)}^{2}]}^{0.5}*\text{BGE}$$

The similarity among 16S rDNA samples was compared at both initial and stationary growth phases using all ASVs. Stationary growth phase samples were organized into groups of phylogenetically similar clusters using the phyloseq-generated weighted UniFrac distance matrix in a similarity profile (SIMPROF) analysis (*p* > 0.01; Primer v6; Clarke et al., 2008). Group significance was determined by permutational multivariate analysis of variance (PERMANOVA) using the ‘adonis’ function in R within the ‘vegan’ package (v2.5–6) with pairwise analysis at 9,999 permutations (Legendre and Andersson, 1999; Oksanen et al., 2015). Unique ‘indicator’ species to each initial and stationary phase were identified based on multi-pattern analysis using the ‘multipatt’ function in R within the ‘indicspecies’ package (v1.7.8) (Dufrêne and Legendre, 1997; De Cáceres and Legendre, 2009). The point biserial correlation coefficient function (‘r.g’ option) and 9,999 permutations of the statistical test identified unique ASVs to the groups of phylogenetically similar samples.

Shannon-Wiener *H* index values and their associated errors were estimated using PAST software (v4.03). Values and trends in the *H* index did not differ significantly between rarified and non-rarified sample sets, suggesting little effects of variable sampling effort on alpha diversity indices (Lande, 1996); as such, diversity values will be presented on non-rarified datasets. The significance in the Shannon index between two samples was tested in PAST software based on Hutcheson’s *t*-test (Hutcheson, 1970). The DivNet package in R was also used to test for significant differences in Shannon indices across depths and growth phases among all samples, while accounting for unobserved species (Willis, 2019).

## RESULTS

### Experimental Dynamics of Bacterioplankton, DOC and Growth Efficiencies

#### Bacterioplankton –

**FIGURE 1 |:**
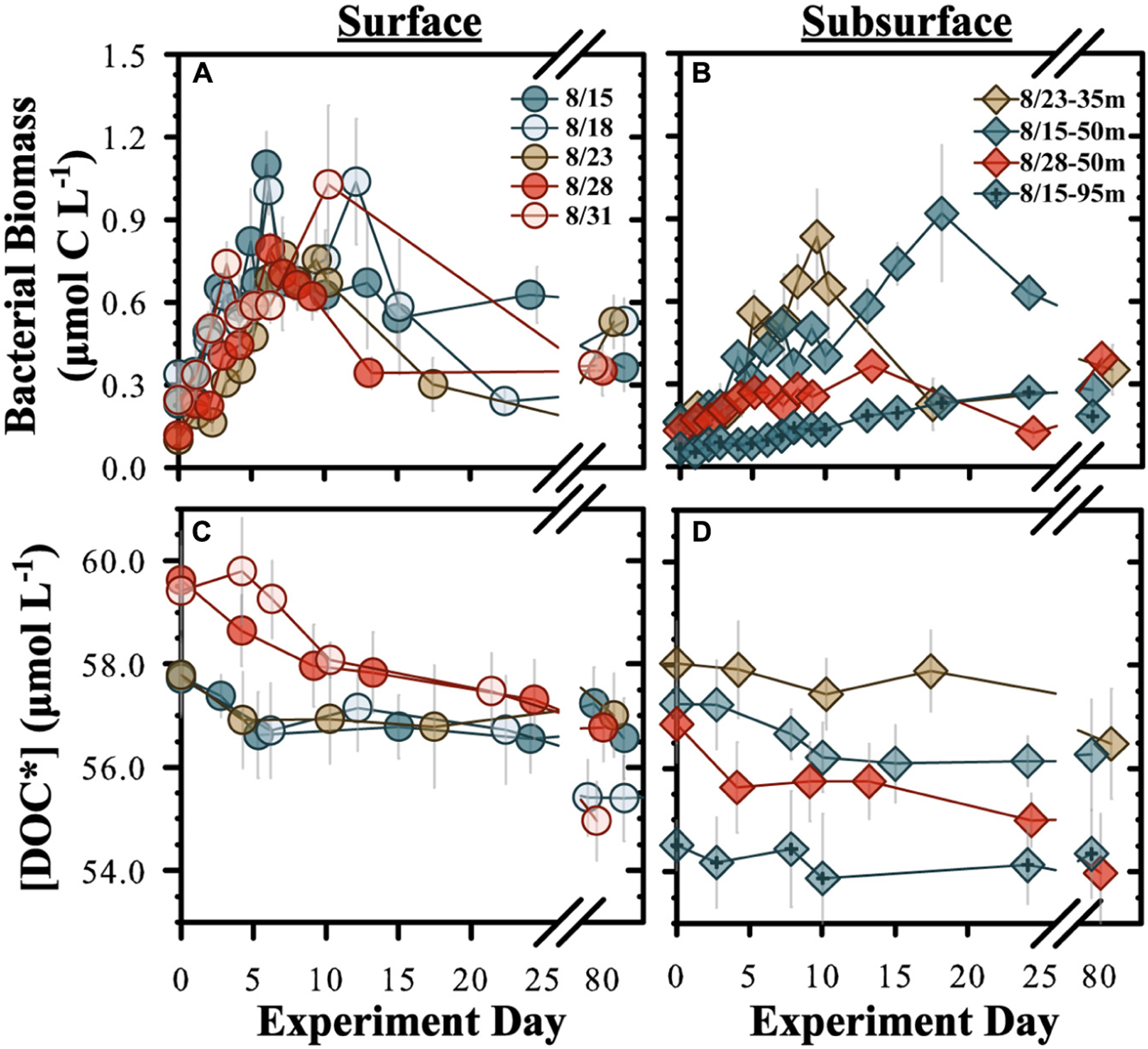
Temporal changes for “diluted” OM remineralization experiment bacterioplankton biomass **(A,B)** and [DOC*] **(C,D)**. “Surface” **(A,C)** refer to experiments conducted from 5 m and “subsurface” **(B,D)** refers to experiments conducted at 35, 50, and 95 m. DOC* denotes that concentrations were corrected for bacterioplankton carbon but contain an unconstrained contribution of C < 3.0 μm. Incubation start dates are given.

BB in *diluted* experiments (70% < 0.2 μm:30% < 3.0 μm) from surface water (5 m) began to increase within 1–2 days after initiation and reached stationary growth phase within 6–10 days, increasing by an average BB of 0.7 ± 0.2 μmol C L^−1^ by stationary phase (Figure 1A and Table 2). The BP rate in surface experiments averaged 0.9 ± 0.4 μmol C L^−1^ d^−1^ but was significantly elevated (two-tailed *t*-test, *p* < 0.05) in the Aug. 15 experiment. The subsurface experiments conducted at 35, 50, and 95 m reached stationary phase within 9–10 days (Figure 1B). The mean BP rates among the subsurface experiments (0.04 ± 0.02 μmol C L^−1^ d^−1^) were significantly (two-sampled *t*-test, *p* = 0.004, df = 14) lower than the surface mean (Table 2).

*Undiluted* experiments (<3.0 μm filtrate only) were also conducted from the same initial water as for *diluted* experiments but samples were only collected for BB, DOC* and THAA analyses. Estimates of mean BP rates for all surface *diluted* and *undiluted* experiments were statistically indistinguishable (two-sample *t*-test, *p* = 0.54), with pooled means of 0.08 ± 0.03 and 0.10 ± 0.06 μmol C L^−1^ d^−1^, respectively (Table 2 and Supplementary Figure 5). However, the *undiluted* experiments had an earlier and more pronounced decline in BB after reaching stationary growth phase compared with a more stable BB stationary phase in the *diluted* experiments. Both designs included viruses; thus, we interpret the enhanced death phase in the *undiluted* experiments to be a result of grazing pressure.

#### DOC* Concentrations –

Initial [DOC*] for the surface *diluted* experiments ranged between 57.7 ± 0.4 and 59.8 ± 0.6 μmol C L^−1^ (Table 2). Mean short-term DOC* removal rates were significantly (*p* < 0.05) modeled for the Aug. 28 and Aug. 31 experiments, averaging 0.18 ± 0.01 μmol C L^−1^ d^−1^ (*n* = 2). After ~90 days of incubation, the final [DOC*] in surface experiments ranged from 55.0 ± 0.4 to 57.0 ± 0.5 μmol C L^−1^ (Table 2). Among the subsurface experiments, a significant short-term DOC* removal rate of 0.10 ± 0.02 μmol C L^−1^ d^−1^ was only resolvable in the 50 m Aug. 15 *diluted* experiment. Mean short-and long-term DOC* removal rates in the *undiluted* experiments averaged 0.24 ± 0.09 (*n* = 4) and 0.03 ± 0.01 μmol C L^−1^ d^−1^, respectively; these rates were not statistically different from *diluted* experiments (two-tailed *t*-test *p* = 0.54 and *p* = 0.57, respectively). Most *undiluted* subsurface experiments (except at 35 m) exhibited detectable DOC* removal, with a mean short-term DOC* removal rate of 0.16 ± 0.04 μmol C L^−1^ d^−1^ (Table 2).

#### Bacterial Growth Efficiencies –

BGE values were determined by dividing the rate of bacterial production (T0 to stationary) by DOC* removal rates (eq. 3). Mean surface (5 m) BGEs in *diluted* and *undiluted* experiments were an essentially identical 32 ± 8% and 32 ± 9%, respectively. Subsurface experiment BGE values determined using the EXPORTS cell carbon relationship ranged from 19 ± 8% to 35 ± 10% (mean of 28 ± 7%; Table 2).

### THAA Composition

**FIGURE 2 |:**
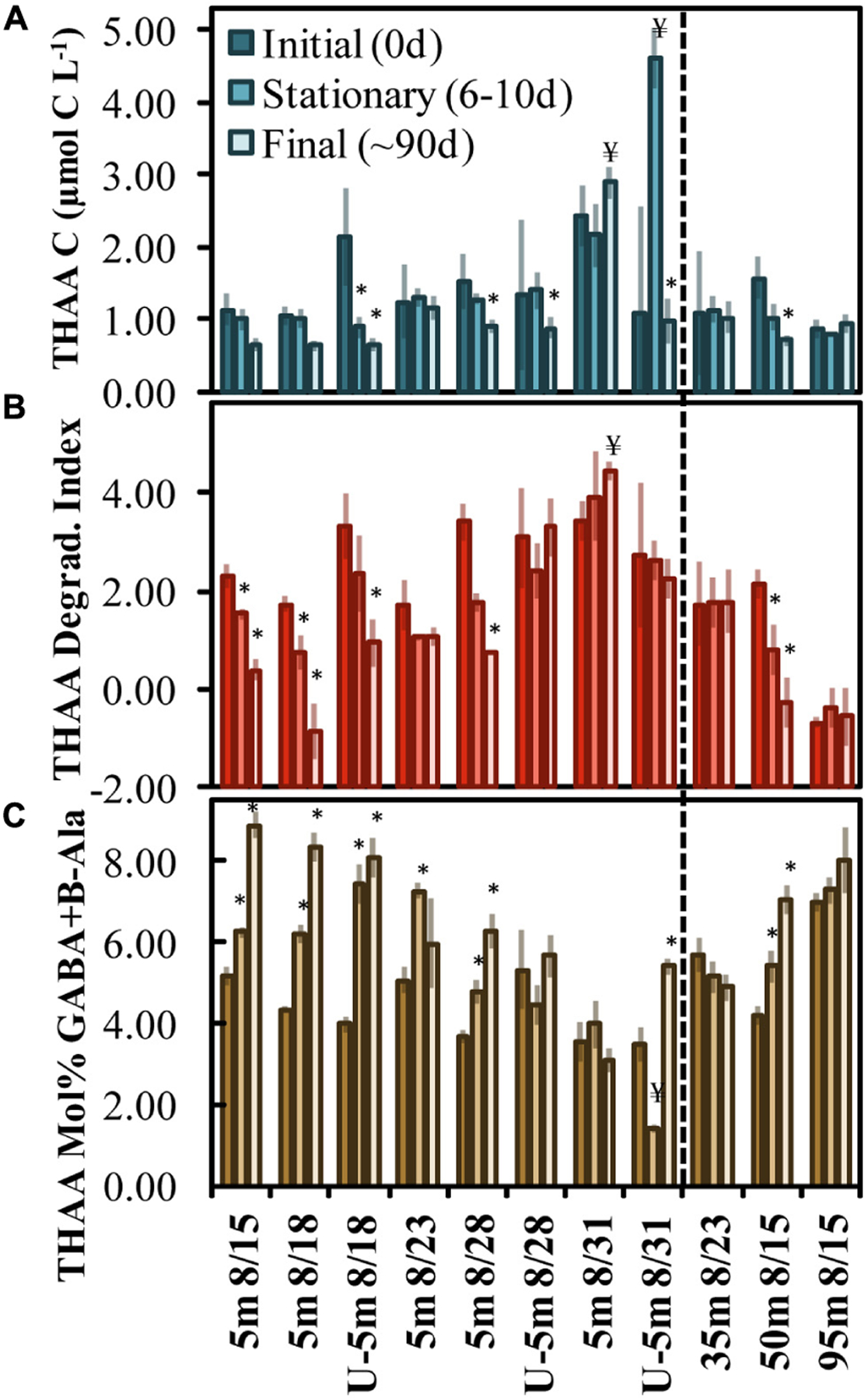
OM remineralization experiment changes in total hydrolyzable amino acid (THAA) C **(A)**, the degradation index score **(B)**, and mol% GABA + B-Ala **(C)** between initial (0 day), stationary phase (6–10 days) and final time points (~90 days). The *x*-axis indicates the experiment initiation date and those beginning with a “U” refer to undiluted experiments (all others are 70% dilutions). The * and ¥ above the bars identify treatments that exhibited a statistically significant (two-tailed *t*-test *p* < 0.05) shift to a more or less diagenetically altered OM composition, respectively. Error bars refer to propagated standard errors. Dashed vertical line differentiates surface from subsurface experiments.

Total hydrolyzable amino acid, expressed in C units, averaged 1.45 ± 0.46 μmol C L^−1^ at T0 across all experiments (Figure 2A and Supplementary Table 1). Only the Aug. 31 experiment was initiated with a THAA concentration (in C units) that was significantly (two-tailed *t*-test, *p* < 0.05) elevated compared to other experiments. THAA C decreased by the final time points in 5 out of the 11 experiments, with an average decrease of 0.65 ± 0.26 μmol C L^−1^. The decrease in THAA C represented 33 ± 11% of the [DOC*] decrease (Figure 2A). A relative decrease in the THAA-based degradation index (DI) and a relative increase in mol% GABA + B-Ala over the incubation period indicated that OM was diagenetically altered to a more degraded state (Cowie and Hedges, 1994; Dauwe and Middelburg, 1998; Davis et al., 2009).

The mol% GABA + B-Ala was initiated with a significantly reduced diagenetic alteration (two-tailed *t*-test, *p* < 0.05) in the Aug. 28 and Aug. 31 experiments, indicating that the OM initial condition was “fresher” compared with other experiments. Comparing the three subsurface experiments, the mol% GABA + B-Ala indicated that the OM was fresher and became significantly altered in the 50 m experiment begun on Aug. 15. Among the surface and subsurface OM remineralization experiments, the DI and mol% GABA + B-Ala values indicated that OM became significantly (two-tailed *t*-test p < 0.05) diagenetically altered with time of incubation in 9 of 11 total experiments (denoted with asterisks above the bars in Figures 2B,C).

### LC-MS/MS

Approximately 1,831 unique molecular features that had an MS/MS spectrum assigned were identified (that were not present in either the processing or instrument blanks) by LC-MS/MS in the PPL-extracted samples. Peaks for all samples were aligned using MZmine (Supplementary Text 1), thereby reducing mass error and improving confirmation of spectral information (Merder et al., 2020). Based on a comparison of fragmentation patterns from aligned spectra in the EXPORTS dataset to that of publicly available MS repositories, 151 unique features matched compounds to an MS library within <0.01 Da mass offset, and an additional 244 library analogs were detected with a mass difference of <50 Da (Supplementary Table 2).

**FIGURE 3 |:**
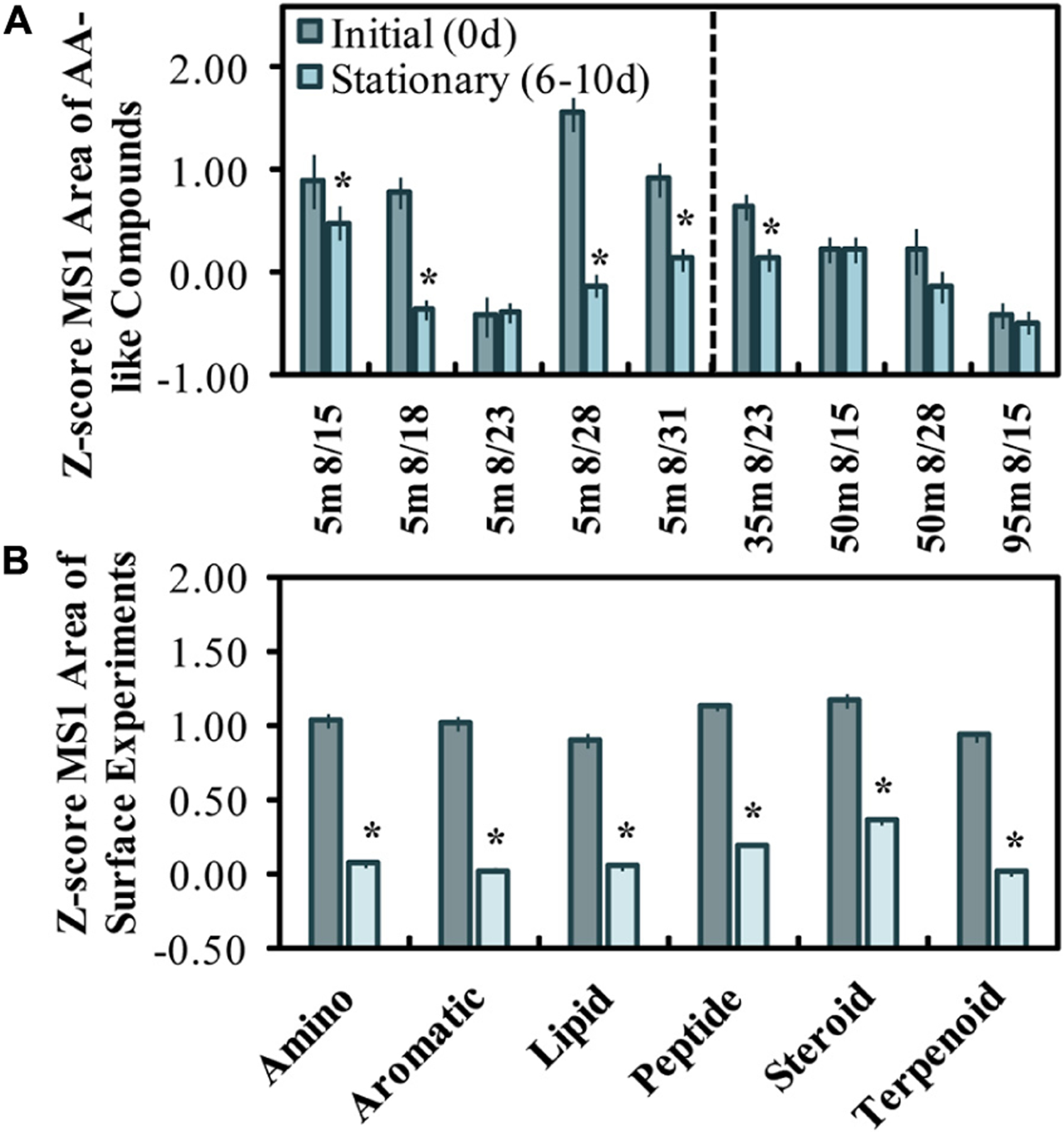
*Z*-scored peak areas of amino acid-like compounds (*n* = 21) in surface experiments determined by LC-MS/MS, where dates on the x-axis refer to the experiment start date and sample depth **(A)**. Mean *z*-score changes by 6 molecular feature classes initially abundant in surface experiments (*n* = 85), excluding the 8/23 experiment **(B)**. Error bars represent standard errors, and the * above the bars refers to a significant (two-tailed *t*-test, *p* < 0.05) decrease between initial and stationary phase. A *z*-score change of −1.0 refers to one standard deviation decrease.

The *z*-scores of LC-MS/MS aligned peak areas determined for initial and stationary phase samples were used to evaluate the change in amino acid-like and other compound classes (Figures 3A,B). The mean *z*-scores of AA-like compounds decreased significantly (*p* < 0.05) in all experiments except for the one initiated on Aug. 23 (Figure 3A). The Aug. 28 experiment had the highest initial *z*-scores and showed the greatest *z*-score change in MS1 peak areas from initiation (Figure 3A). Roughly half of the compounds initially abundant in the experiments were classified either as amino acid-like or terpenoid-like out of six compound classes. A decrease in *z*-scores for the assigned classes (excluding the Aug. 23 experiment) suggests that a range of compound class types (i.e., amino acids, lipids, steroids, terpenoids) was reduced or altered by stationary growth phase in most surface experiments.

### 16S rDNA Community Composition

**FIGURE 4 |:**
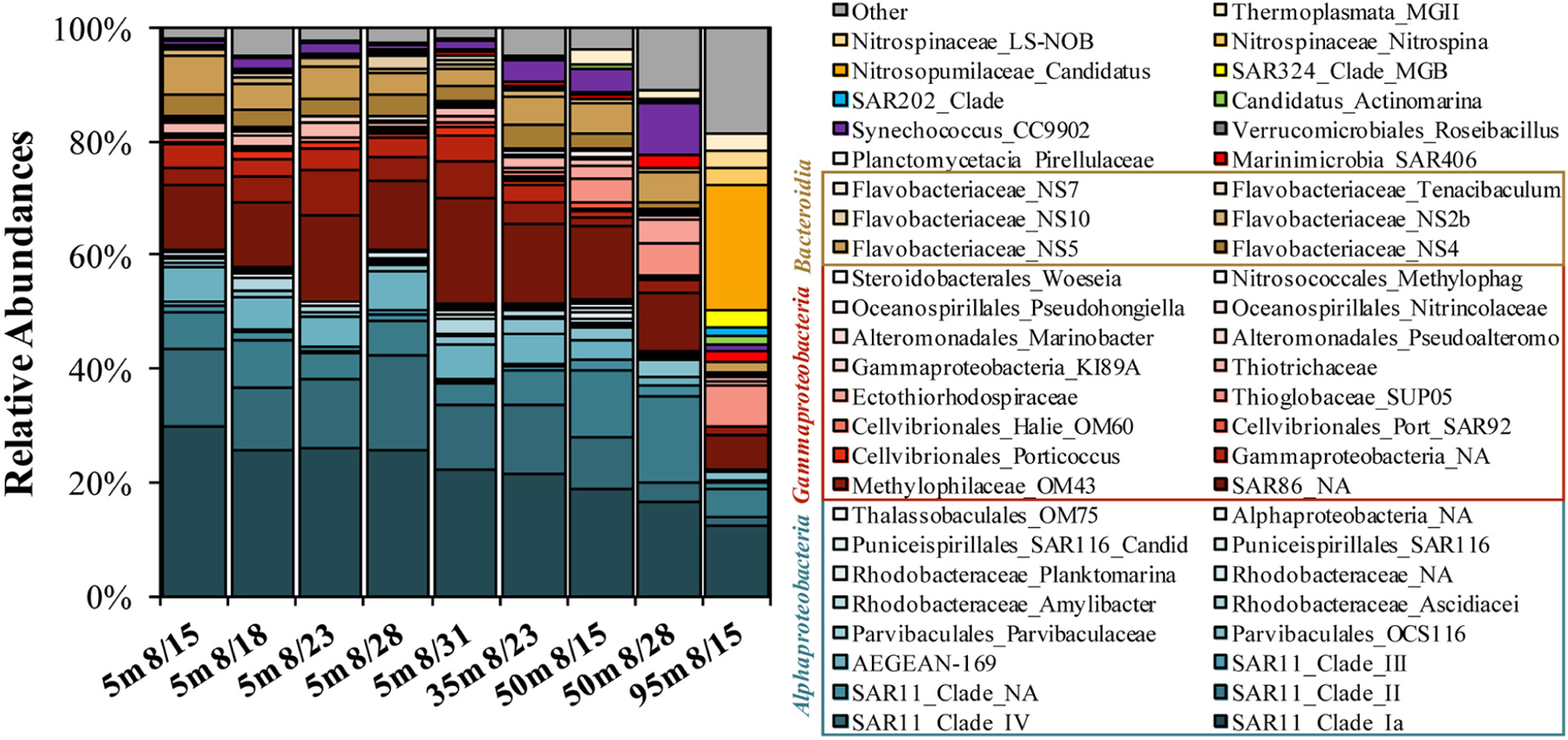
Stacked bar plot of top 50 most abundant genera determined on 16S rDNA amplicons as observed at the initial condition of the OM remineralization experiments. Both family and genus level names are included in the legend, where available.

The 16S DNA amplicons for the initial condition of surface experiments were dominated by the *Alphaproteobacteria* SAR11 Clades Ia, II, and IV (combined relative abundances of 37–50%), with minor contributions by the *Gammaproteobacteria* SAR86 (12–19%) and the *Bacteroidetes Flavobacteriaceae* NS4, NS5, and NS2b (7–12%; Figure 4). The 50 m sample collected on Aug. 28 contained elevated contributions of *Synechococcus* CC9902 (9.1%) and the *Gammaproteobacteria Thioglobaceae* family SUP05 Clade (6%). The 95 m sample was initially elevated in the *Thaumarchaeota* genus *Candidatus Nitrosopumilus* (22%) and had other unique contributions by the *Deltaproteobacteria* SAR324 Clade (3%) and the *Chloroflexi* SAR202 Clade (2%).

**FIGURE 5 |:**
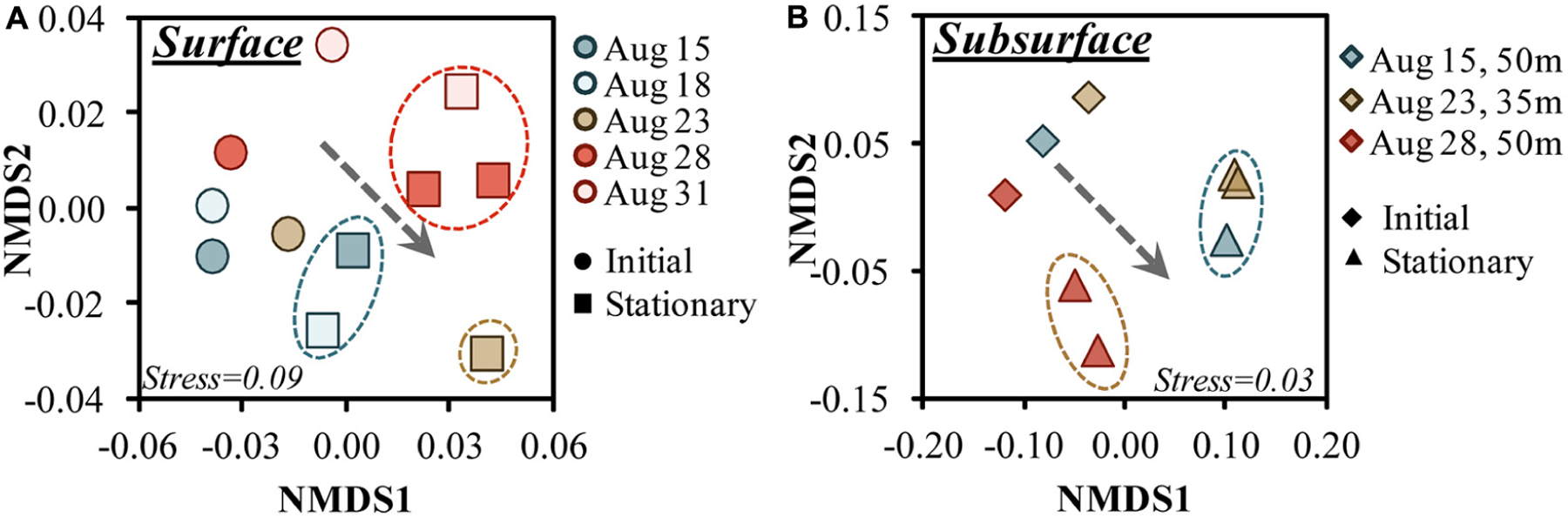
Non-metric multidimensional scaling (nMDS) ordination plots of the 16S rDNA amplicon sequence variants for the surface **(A)** and subsurface **(B)** OM remineralization experiments between initial and stationary growth phase. Circles around stationary phase samples identify those samples that grouped significantly (*p* > 0.01) based on SIMPROF analysis (Clarke et al., 2008); the dotted arrow illustrates the general trend between initial and stationary growth phases toward the positive nMDS1 region. Note that the 95 m communities, though available, were not included in the subsurface figure as it would have obfuscated initial/stationary trends shown for other depths.

nMDS analysis of the 16S amplicons ordinated the communities by growth phase in both surface and subsurface experiments (Figures 5A,B). Both surface and subsurface experiments transitioned to significantly different communities (PERMANOVA for surface *R*^2^ = 0.384, *p* = 0.009) by stationary growth phase (Figure 5A). By stationary phase, the bacterioplankton formed three main groups (SIMPROF *p* > 0.01), where the Aug. 23 experiment did not group with any other stationary phase surface experiment (Figure 5A). Among the 35 and 50 m samples, the 50 m Aug. 28 experiment exhibited a significantly unique stationary phase relative to the other samples (Figure 5A).

The Shannon-Wiener *H* index increased significantly (Hutcheson’s *t*-test, *p* < 0.01) between initial and stationary growth phases in five out of nine experiments (Table 3), and *H* index values also increased significantly (*p* < 0.05) as a function of depth (Willis, 2019). The *H* index values ranged 3.2–3.9 (Table 3). Among the 5 m experiments, the greatest changes were observed in the Aug. 18 experiment, exhibiting an 11% decrease in the *H* Index and an 11% increase in the Aug. 23 experiment.

**FIGURE 6 |:**
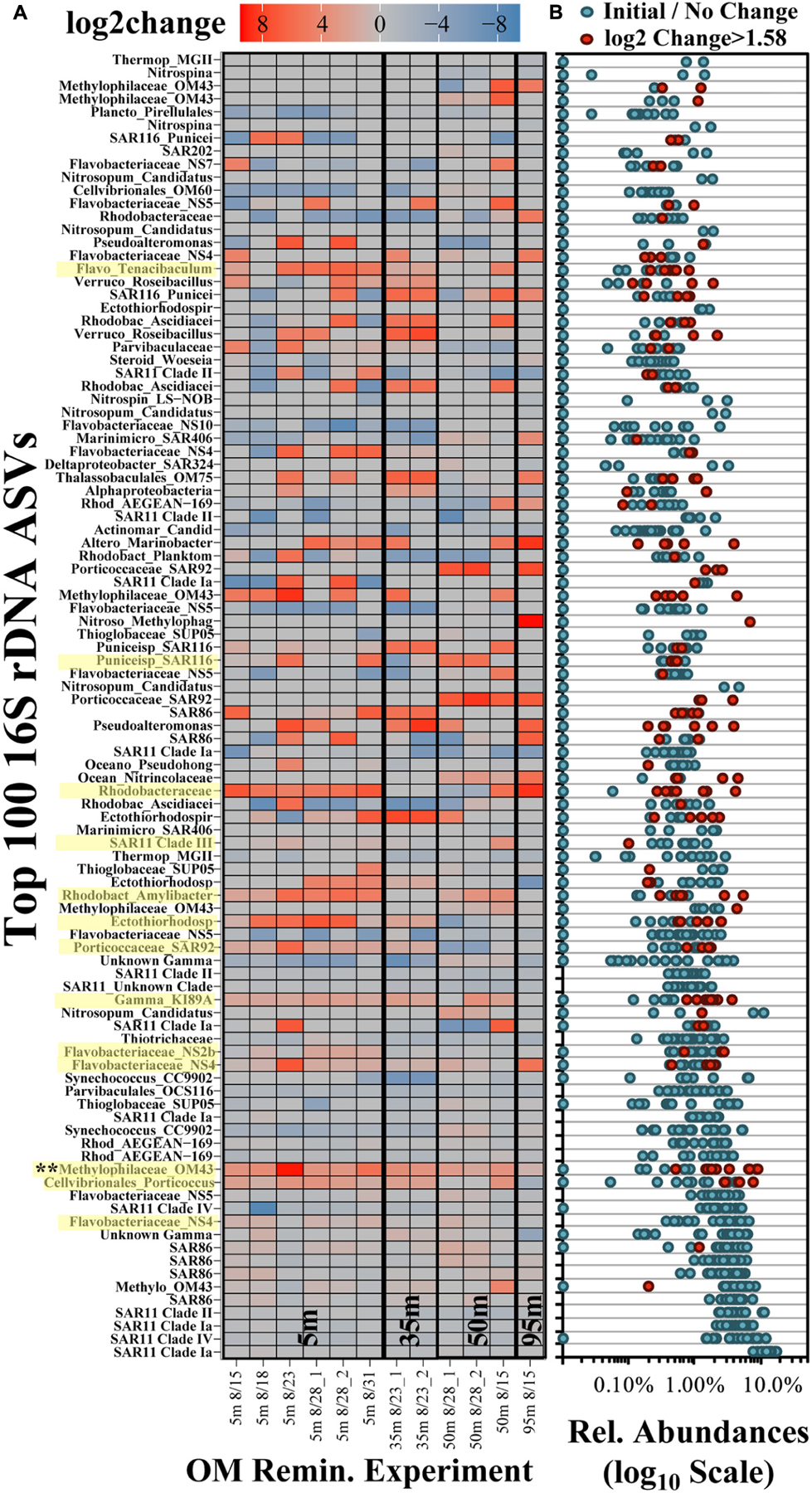
Log2-fold change heatmap of the 100 most frequent ASVs in the OM remineralization experiments **(A)** and the associated relative abundances **(B)**. The *x*-axis in **(A)** refers to the date of experiment initiation and the appended number in the sample names refers to duplicate treatment values, where available. ASVs highlighted in yellow on the *y*-axis identify those that were significantly (*p* < 0.05) elevated and determined to be indicators in surface experiments (De Cáceres and Legendre, 2009). Each dot in **(B)** represents the abundances associated with the samples presented in **(A)**; red dots identify ASVs with log2-fold change > 1.58 (equivalent to a threefold difference) and blue dots refer to all other samples.

Across all surface and subsurface experiments, 100 ASVs accounted for an average of 96.0 ± 4.3% (range of 84.0–99.1%) of the total relative abundance of ASVs (Figure 6). Many of the most abundant ASVs (up to the top 13 ASVs, predominantly the *Alphaproteobacteria* SAR11 and *Gammaproteobacteria* SAR86) did not exhibit significant log2-fold increases between initial and stationary growth phases and comprised an average of more than 60% of the total surface relative abundances (Figure 6B). Though lower in relative abundances, 54 of the 100 most abundant ASVs exceeded a log2-fold increase of 1.58 (equivalent to a threefold difference; Wear et al., 2018). The red colors in the heatmap in Figure 6A identify log2-fold increases and indicate growth of that ASV by stationary growth phase. Blue colors identify a log2-fold decreases and ASV displacement but by mechanisms that are more difficult to infer; for instance, a negative log2-fold change could signify that either ASV populations remain the same while others grow, or it could represent a preferential loss of those ASVs.

Multi-pattern analysis identified 13 ASVs that were significantly unique (*p* < 0.05) in surface stationary phase samples compared with the initial composition (highlighted yellow in Figure 6A; Dufrêne and Legendre, 1997; De Cáceres and Legendre, 2009). Significantly unique responding ASVs were associated with the *Methylophilaceae* Clade OM43, the NS4, NS2b and *Tenacibaculum* genera of the *Flavobacteriaceae* family, *Cellvibrionales Porticoccus*, members of an unclassified genera and the *Amylibacter* genera of the *Rhodobacteraceae* family, the SAR116 family of the *Puniceispirillales* order, and an unclassified *Ectothiorhodospiraceae* genus (among others highlighted in Figure 6A). Other taxa that exhibited pronounced but variable responses included those associated with the *Marinobacter* genus of the *Alteromonadales* order and the *Sulfitobacter* genus of the *Rhodobacteraceae* family. Of the ASVs significantly unique to stationary phase, an ASV associated with the *Methylophilaceae* Clade OM43 exhibited a significant increase (*p* < 0.05) in experiments from both surface and subsurface depths. Taxa increasing only in the 95 m experiment included those within family *Methylophagaceae* of the *Nitrosococcales* order, a member of the AEGEAN-169 family and the *Marinimicrobia* Clade SAR406.

## DISCUSSION

The microbe-molecule interaction is one of the most fundamental reactions within the global carbon cycle (Kujawinski, 2011). DOM remineralization experiments assess interactions between natural bacterial assemblages and bulk DOM, with dilution experiments releasing bacteria from top-down mortality associated with bacterivory (Ammerman et al., 1984; Carlson and Hansell, 2015). Results from DOM remineralization experiments can also ultimately be used to expand the utility of field measurements; for instance, the derived growth efficiencies can be applied to proxies of BP (i.e., ^3^H-Leu incorporation rates) to estimate bacterial carbon demand (BCD). Previously applied to OSP, a combination of BP and derived BGEs demonstrated that BCD can, at certain times of the year, exceed rates of primary production (Kirchman et al., 1993; Sherry et al., 1999). Thus, experiments of BP at OSP have demonstrated the significant contribution of bacterioplankton to C cycling.

The OM remineralization experiments conducted at OSP for 5, 35, 50, and 95 m depths estimated an average short-term DOC* removal rate of 0.19 ± 0.08 μmol C L^−1^ d^−1^ (Table 2). Mean removal rates reflect those inferred for a broad class of labile DOM compounds remineralized by marine heterotrophic microbes (Carlson and Ducklow, 1995; Hansell, 2013; Carlson and Hansell, 2015). Average initial [DOC*] values for *diluted* surface OM remineralization experiments were 58.5 ± 1.0 μmol C L^−1^ and after 90 days [DOC*] reached a minimum of 55.0 ± 0.4 μmol C L^−1^. Measured minimum [DOC*] were reflective of previously detected minimum surface [DOC] measured during winter at OSP (Bif and Hansell, 2019). Despite a relatively low variability in initial [DOC*], short-term remineralization rates had a significant direct correlation with initial [DOC*] (*R*^2^ = 0.97; *p* = 0.0026; Table 2). Additionally, the lowest final [DOC*] was associated with highest initial [DOC*] and elevated short-term DOC* removal rate (Aug. 31 in Table 2), suggesting that additions to the DOM pool had a priming effect that ultimately led to enhanced removal of surface accumulated DOC (Carlson et al., 2002; Guenet et al., 2010).

### Elevated Bacterial Growth Efficiencies Detected at OSP

Bacterial growth efficiencies from all depths and experiments ranged 19 ± 8% to 45 ± 13% with a mean of 31 ± 9% (Table 2), based on a relationship between bacterioplankton biovolume (0.03–0.09 μm^3^ cell^−1^) and cell C (~7–18 fg C cell^−1^) determined from samples collected during the EXPORTS study (Supplementary Figure 4). Using the findings here and from previous studies of bacterioplankton cell biovolume and cell carbon (e.g., Gundersen et al., 2002), we assume that the power-law relationship can be applied across the full range of potential cell biovolumes to estimate cell carbon. In addition to the cell carbon relationship derived in the current study, estimates of the bacterioplankton carbon conversion factor were also used to estimate BB using two previously published relationships [cell carbon = 108.8* cell biovolume^0.898^ from Gundersen et al. (2002) and cell carbon = 103.02* cell biovolume^0.59^ from Malfatti et al. (2010)] (Table 2 and Supplementary Figure 4). Based on those relationships from open ocean and coastal sites, the derived carbon conversion factors ranged between 4–10 and 11–22 fg C cell^−1^, respectively (Gundersen et al., 2002; Malfatti et al., 2010).

The use of literature derived cell carbon relationships would either reduce mean BGEs by 12% or increase them by 17%, respectively, compared with the relationship derived in the current study (Table 2). However, even accounting for uncertainty with the carbon conversion factors, the range in BGEs estimated here is most reflective of those previously measured during summer months at OSP (~40 ± 15% in summer vs. ~10 ± 5% in spring; Sherry et al., 1999). In a global comparison, these summer BGE estimates at OSP also fall within the upper quartile of median open ocean values (median of ~22%, upper quartile of ~34%) estimated in a review of BGEs from a range of freshwater and marine sites (del Giorgio and Cole, 1998).

Organic matter remineralization experiments of similar design show that low BGEs (4–12%) are generally associated with a more bioavailable DOM and with additions of sugars (Carlson and Ducklow, 1996; Nelson et al., 2013). A relatively low removal of 1–2 μmol C L^−1^ (over ~7 days), similar to that observed at OSP (Table 2), has also been associated with elevated BGEs of 30–50% (Carlson et al., 1999; Halewood et al., 2012; Wear et al., 2015). Kirchman (1990) demonstrated that bacterioplankton at OSP had consistently elevated growth when amended with amino acids as compared with sugar amendments, suggesting that the quality of OM plays an important role in controlling the production and (presumably) BGEs. Given influences of AAs on bacterioplankton (Kirchman, 1990), the empirically derived BGEs for OSP presented here and in Sherry et al. (1999) may be associated with periodic elevation in contributions of hydrolyzable amino acids.

In the context of iron limitation in the NE Pacific (Martin and Fitzwater, 1988), previous estimates of iron quotas for bacteria at OSP suggested that estimated BGEs (12–23%) were reduced compared with other regions due to local iron limitations to bacterial growth (Tortell et al., 1996). This hypothesis is consistent with other findings that BGEs can become significantly reduced (e.g., from 60% down to 15%) in iron-limited regions of the South Pacific (Baltar et al., 2015). Though not explicitly tested in the current study, the relatively high BGEs presented here imply that iron may not have been limiting to bacterioplankton growth during the cruise or under the experimental conditions.

It is important to also note that a greater number of *undiluted* experiments had resolvable DOC* remineralization rates and BGE values (Table 2; 7 *undiluted* vs. 3 *diluted* experiments). This outcome may have been in part due to elevated contributions from a more labile OM within the 0.2–3.0 μm range or due to elevated initial BB in the *undiluted* treatments (Supplementary Figures 5a,b; Zehr et al., 2017); alternatively, grazer interactions led to an enhanced remineralization of OM and release of inorganic nutrients (Taylor et al., 1985). It is unclear which factor(s) was most influential in generating the greater number of experiments with detectable DOC* removal in the *undiluted* treatments. Despite the greater number of resolvable rates and BGEs in the *undiluted* experiments, the average BGEs and associated short-term BP and DOC* remineralization rates from *diluted* and *undiluted* experiments were not significantly different (two-tailed *t*-test, *p* > 0.05), so changes in OM quality from the two experiment types will be presented together to infer connections between OM bioavailability and OM quality.

### OM Composition Changes in Experiments

#### Hydrolyzable Amino Acids as Indicators of OM Bioavailability

Indices inferring the degradation state of OM, like the THAA-based DI score presented here, are useful proxies for OM diagenesis (Cowie and Hedges, 1994; Dauwe and Middelburg, 1998; Davis et al., 2009; Kaiser and Benner, 2009). Such indices do not estimate the rate of C removal, nor can they estimate how much of the accumulated OM pool is bioavailable. However, previous studies using remineralization experiments similar to those presented here have linked the initial THAA- or aldose-based indicators of degradation state to OM bioavailability (Nelson et al., 2013; Shen and Benner, 2019), where results suggest that a more bioavailable OM can be associated with an OM composition that is relatively less diagenetically altered.

**FIGURE 7 |:**
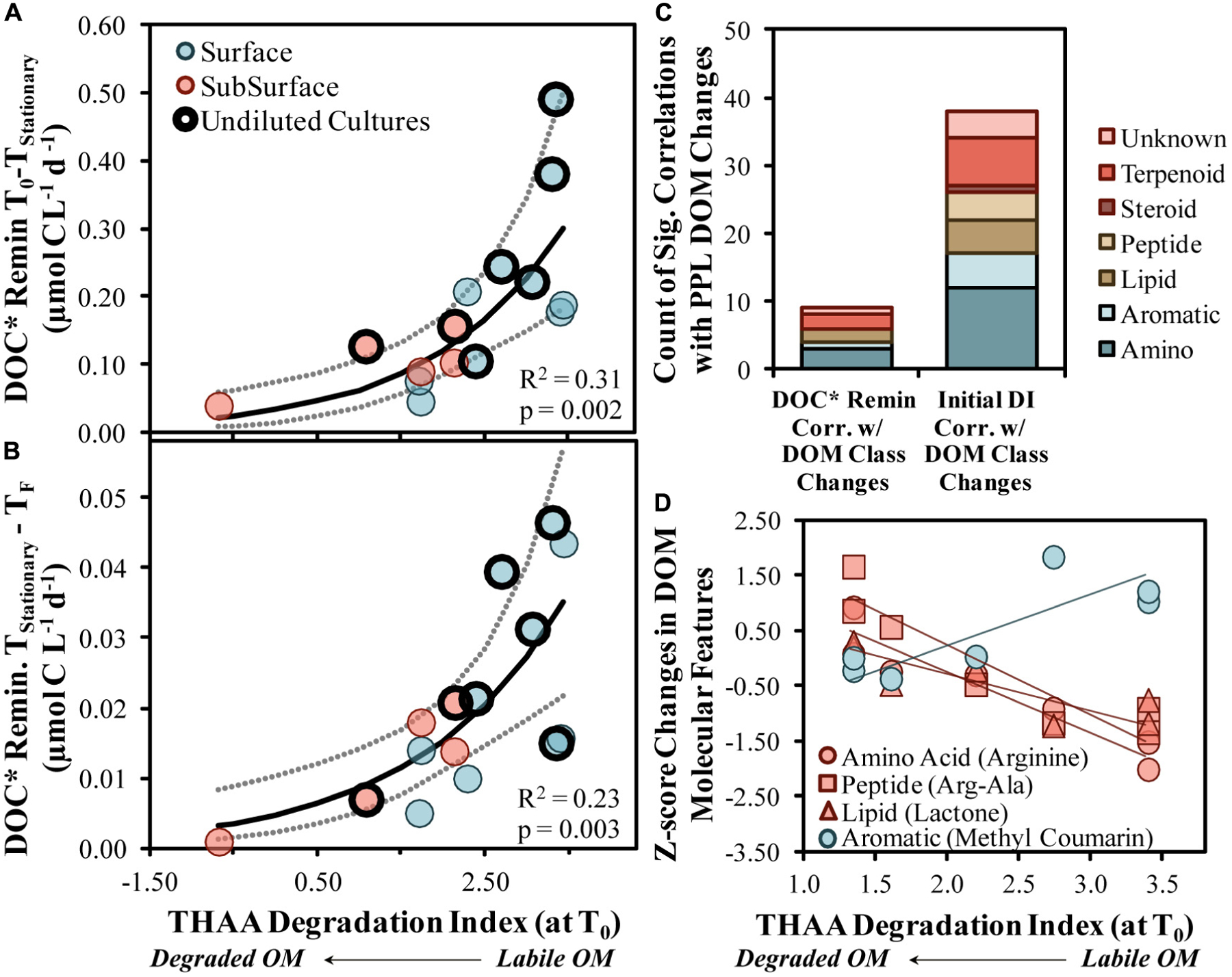
Short- **(A)** and long-term **(B)** DOC* drawdown in OM remineralization experiments compared with the initial THAA-based degradation index (DI) score. Model II non-linear fits are represented by solid lines while dotted lines surrounding these fits represent the ±95% confidence intervals. Counts of compound class changes in MS1 peak area between initial and stationary phases that have significant (*p* < 0.05) correlations with either the short-term DOC* remineralization rate or the initial THAA-based DI **(C)**. Four example scatter plots between the initial THAA-based DI and z-score changes are shown in **(D)**. A *z*-score change of −1.0 refers to one standard deviation decrease for that compound. DOC* denotes that concentrations were corrected for bacterioplankton carbon but contain an unconstrained contribution of C < 3.0 μm.

DOC* drawdown rates increased exponentially as the initial DI score increased, suggesting that, when comparing among this set of experiments, the initial DI score was an adequate predictor of both short- and long-term DOC* drawdown rates (*R*^2^ = 0.31 with *p* = 0.002 and *R*^2^ = 0.23 with *p* = 0.003, respectively; Figures 7A,B). These relationships infer that a less diagenetically altered, more labile OM corresponded with an increased DOC* remineralization rate and, thus, increased OM bioavailability. The significant positive correlations presented in Figures 7A,B also provide support to the hypothesis that marine BP is largely controlled by bottom-up carbon availability in the form of OM (Ducklow, 1992).

THAA concentrations and DI scores presented here (Figures 2A,B) are at the upper end of surface ocean DOM collected near Hawaii and Bermuda and DOM produced from cultures of phytoplankton and bacteria (THAA of 0.8–1.5 μmol C L^−1^ and DI of 1–4; Kaiser and Benner, 2009). The comparison with other marine AA data implies that a portion of the surface OM collected from OSP in August 2018 was recently produced. Additionally, though THAA C values were relatively similar at 95 m, the significant shift down in DI Score is similar to findings with depth near Hawaii (Kaiser and Benner, 2009), suggesting the OM was diagenetically altered by 95 m.

#### LC-MS/MS Supports and Expands OM Composition Changes

High-resolution mass spectrometry approaches to studying marine OM continue to evolve with technological developments (Moran et al., 2016), where the use of LC-MS/MS has recently emerged as a potential non-targeted approach to identifying putative library matches of specific molecular compounds (Longnecker et al., 2015). When aligned spectra (Merder et al., 2020) are used with molecular networking approaches (Aron et al., 2020), hundreds of unique molecular features can be classified at the compound level (Longnecker and Kujawinski, 2017; Petras et al., 2017). Samples analyzed by LC-MS/MS presented here suggest that putative amino acid-like and other compound classes were modified by bacterioplankton in most OM remineralization experiments (Figure 3B), consistent with the mol% GABA + B-Ala trends from the more quantitative HPLC-based amino acid analysis (Figure 2C). In addition to the consistent modification of relevant compounds, several compounds with peak area reductions correlated with both the initial DI score (*n* = 38 compounds) and the short-term DOC* remineralization (*n* = 9 compounds) rate (Figures 7C,D). About 20–25% of the correlated compounds were within the amino acid class of compounds, agreeing with the finding that THAA C represented about 30% of the DOC* removed in experiments (Figure 2A).

Enhanced removal of amino acids (arginine), proteins (argala) and the lipid quorum sensing compound *N*-octanoyl-l-homoserine lactone were associated with initially more labile OM (Figure 7D). Quorum sensing molecules detected in marine environments have typically been associated with particles and/or particle-seeking bacterioplankton, the presence of which can increase enzymatic activity and OM degradation rates (e.g., Hmelo et al., 2011). A reduction of these compounds in the experiments was accompanied by an increase in quorum sensing inhibitors like coumarin and cinnamic acid, which could be a competitive-based protective mechanism initiated by certain heterotrophic bacteria, like *Gammaproteobacteria* (Chen et al., 2019). The elevation of inhibitors in experiments with more labile OM, perhaps primarily in the dissolved phase, could have encouraged competition between taxa, thereby enhancing community diversity.

Other recent studies have employed a similar untargeted LC-MS/MS analysis to evaluate influences of microbial activities on the composition of PPL solid-phase-extracted DOM. For instance, one of the first marine DOM studies to use this untargeted molecular networking approach from diatom cultures found several physiologically relevant lipids produced by diatoms (Longnecker and Kujawinski, 2017). Another study identified a significant release of peptides by viral lysis of *Synechococcus* as compared to exudation or contained with cells, identifying an important source of N to marine systems (Ma et al., 2018). We found significant relationships between the DI score and peptides and lipids (Figures 7C,D), where OSP is often dominated by small (<5 μm) phytoplankton cells (Boyd and Harrison, 1999), potentially also serving as an important OM source in our experiments. Another study of temperate lake DOM indicated elevated microbial community richness to be associated with a more complex DOM (Muscarella et al., 2019), demonstrating promise in bringing together complex microbial community interactions and the untargeted OM characterization approach.

While trends were observed across experiments here, we must emphasize that (1) PPL cartridges used to isolate marine DOM samples only retain up to 60% of the organic carbon from the initial sample (Dittmar et al., 2008; Petras et al., 2017), (2) the LC-MS/MS does not effectively ionize certain compound classes, including neutral sugars (Johnson et al., 2017), and (3) the complexity of DOM presents unique challenges to effectively separating compounds during LC-MS/MS analysis (Hawkes et al., 2018). Thus, the changes in LC-MS/MS compounds highlighted here reflect relative differences among the samples analyzed. Despite potential limitations due to the selected extraction and ionization methods, the correlations between putative compounds and OM bioavailability indicators demonstrate that the method can identify changes to compounds relevant for microbial processes.

### Community Composition Connections With OM Bioavailability

**FIGURE 8 |:**
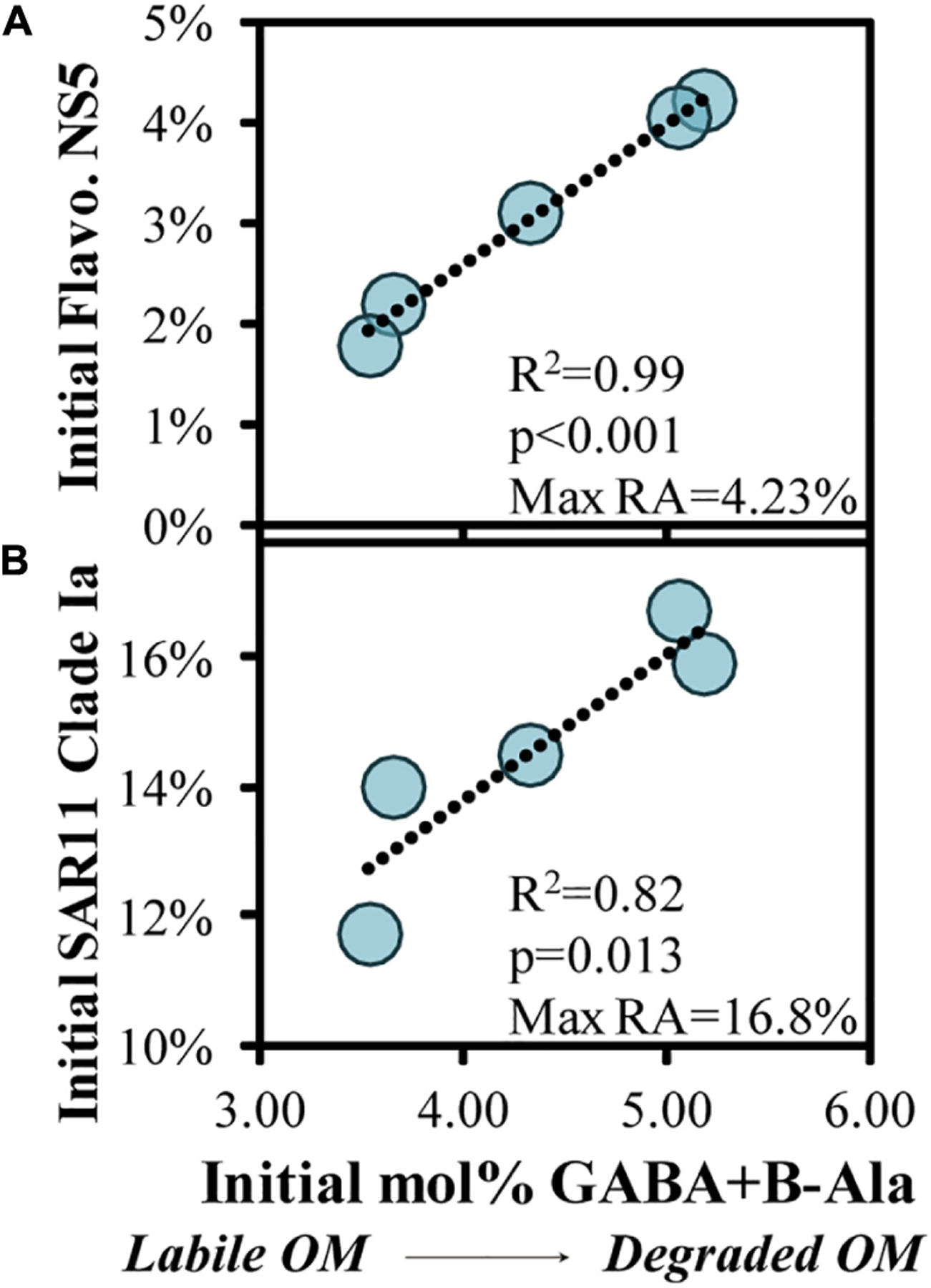
Significant (*p* < 0.05) correlations between the initial THAA-based mol% GABA + B-Ala and initial relative abundances of an ASV within the Flavobacteriaceae NS5 **(A)** and SAR11 Clade Ia genera **(B)**, collected in the surface OM remineralization experiments. The SAR11 Clade Ia in **(B)** was the most abundant ASV among the surface experiments. Noted within each figure are the maximum relative abundances (RA) detected for each ASV.

Quality of the source OM influences the community composition structure, and vice versa, in both field and experimental studies (Nelson et al., 2013; Wemheuer et al., 2014; Díez-Vives et al., 2019; Schada von Borzyskowski et al., 2019; Liu et al., 2020; Reji et al., 2020). To assess potential associations of OM indicators with the initial bacterioplankton community, we correlated OM bioavailability indicators with the relative abundances of the top 100 ASV’s, finding two significant correlations (Figure 8). There was a significant positive correlation (*p* < 0.05) between initial abundances of one of the most abundant ASVs, SAR11 Clade Ia, and another abundant ASV, *Flavobacteriaceae* NS5, and the initial mol% GABA + B-Ala (Figures 8A,B).

When the initial DOM was more diagenetically altered, there was a greater fraction of the bacterioplankton community comprised of SAR11 Clade Ia and *Flavobacteriaceae* NS5. The relationship in Figure 8B in particular is counterintuitive given that members of the SAR11 Ia subclade can compete for labile DOM and contribute up to 20% of detected ASVs under oligotrophic conditions (Carlson et al., 2009; Giovannoni and Vergin, 2012; Vergin et al., 2013; Giovannoni, 2017). Thus, it is unlikely that these dominant surface clades are consuming recalcitrant DOM during more oligotrophic conditions, but rather more likely that the relative contribution of these stable taxa become accentuated as other competing taxa become limited and decline in abundance. Numerous factors make predicting DOM transformations based on taxa within the initial conditions tenuous; thus, we instead turn to evaluating the taxa that respond when grown in the dark on naturally occurring OM. While the decreasing log2-fold changes in experiments can be difficult to interpret (as for SAR11 Ia in Figure 6), an increasing log2-fold is most likely due to an increase in the relative abundance of a particular taxa rather than a loss of all other taxa. Thus, microbial and OM dynamics in these incubations are used here to establish connections between specific responding bacterioplankton, OM composition and its subsequent transformation (Carlson and Hansell, 2015).

**FIGURE 9 |:**
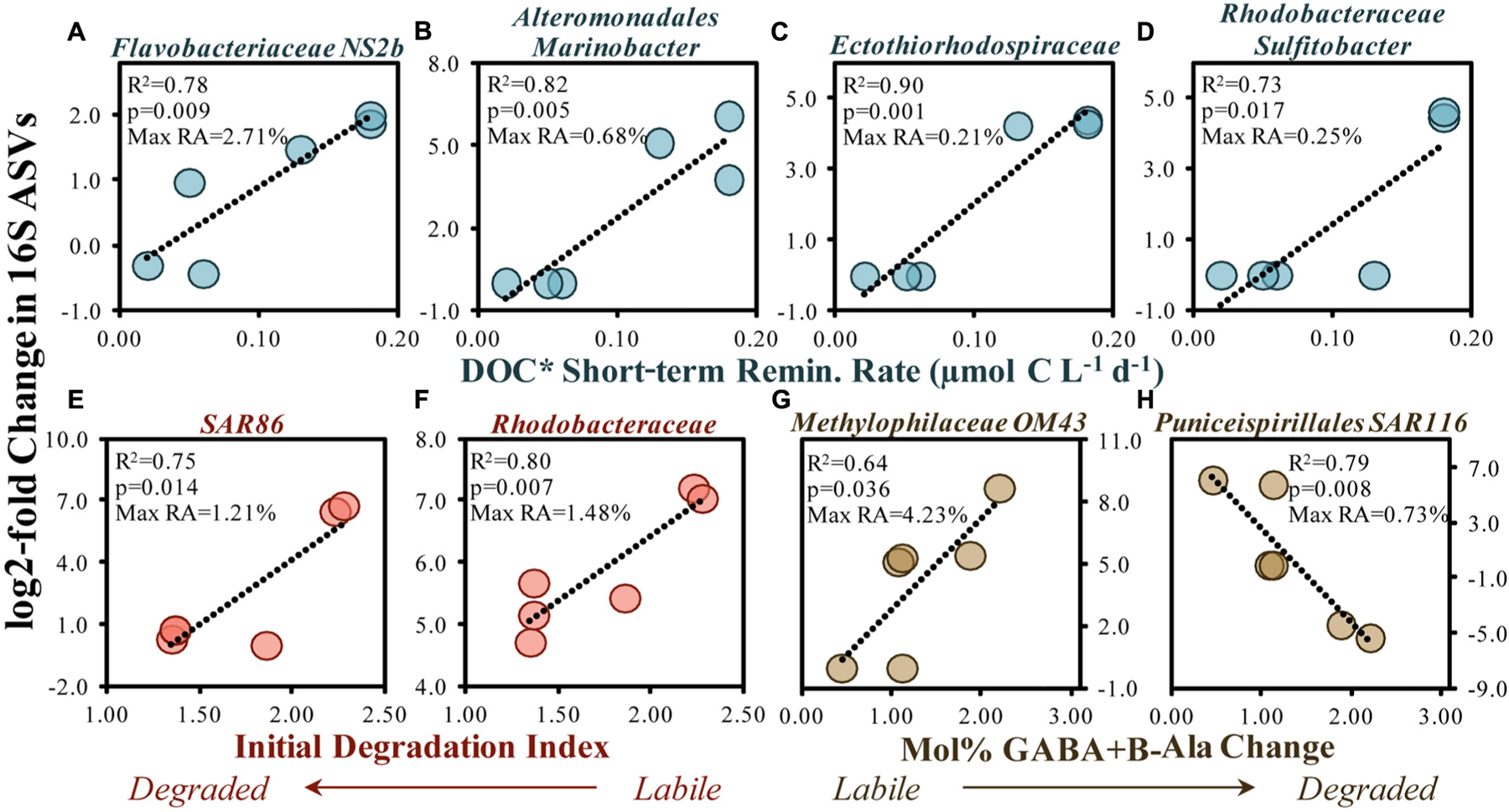
Significant (*p* < 0.05) correlations between the 16S rDNA ASV log2-fold change (between initial and stationary growth phase) and the short-term DOC* remineralization rate **(A-D)**, the initial THAA-based degradation index **(E,F)** and change in the mol% of GABA + B-Ala between initial and stationary growth phases **(G,H)**. Noted within each figure are the maximum relative abundances (RA) detected for each ASV. DOC* denotes that concentrations were corrected for bacterioplankton carbon but contain an unconstrained contribution of C < 3.0 μm.

One of the most frequently responding taxa among surface and subsurface remineralization experiments included an ASV within the OM43 genus of the *Methylophilaceae* family (Figure 3A). As a streamlined methylotroph, OM43 specializes in the removal of C1 compounds (Halsey et al., 2012; Giovannoni et al., 2014), likely able to cleave methyl groups from more complex compounds like polysaccharides (McCarren et al., 2010; Sosa et al., 2015; Gifford et al., 2016). In addition to consistently increasing in abundance in experiments presented here, one member of the OM43 genus also exhibited a positive relationship between log2-fold increases and a change in mol% GABA + B-Ala (i.e., more degraded OM) during experiments (Figure 9G), suggesting that members of this clade may, in part, contribute to the modification of accumulated OM toward a degraded state.

The second most frequent responder in the remineralization experiments (excluding the 95 m experiment), the *Gammaproteobacteria* KI89A, increased in abundances in most treatments and depths (Figure 3A). KI89A has exhibited positive responses to NH_4_^+^ amendments (Goldberg et al., 2017), has elevated protein utilization compared with other groups (Orsi et al., 2016), and elevated abundances associated with the NH_4_^+^-oxidizing *Thaumarchaeota* (Reji et al., 2019). Together, past studies suggest this group to favor N-rich compounds, consistent with amino acid removal in the present experiments.

Other responding ASVs from surface remineralization experiments were correlated with OM bioavailability indicators (Figure 9). For instance, an increased DOC* drawdown rate was significantly (*p* < 0.05) and positively correlated with elevated log2-fold relative abundance increases in members of the *Flavobacteriaceae* NS2b genus, the *Alteromonas Marinobacter* genus, an unknown member of the *Ectothiorhodospiraceae* family, and the *Rhodobacteraceae Sulfitobacter* genus (Figures 9A–D). Greater log2-fold increases were also observed when the initial DI and mol% GABA + B-Ala changes suggested a more labile OM, including SAR86, *Rhodobacteraceae* and the *Puniceispirillales* order (SAR116 family). Several studies have demonstrated that members of these clades respond to recent OM production following phytoplankton bloom conditions (Abell and Bowman, 2005; Teeling et al., 2016; Díez-Vives et al., 2019; Reji et al., 2020) and can be enriched on sinking particles (Duret et al., 2019).

Taxa within the *Flavobacteriales* (NS2b genus), *Alteromonadales (Marinobacter* genus) and *Rhodobacterales* orders are capable of removing a range of compound types including organic acids, sugars, lipids, chitin, proteins and cellulose (Kirchman, 2002; Handley and Lloyd, 2013; Pujalte et al., 2014), and as such may have each contributed to the removal of the diverse classes of compounds associated with the conditions of elevated OM bioavailability (Figures 7C, 9A,B,F). *Rhodobacteraceae Sulfitobacter*, though low in total relative abundances (up to 0.25% relative abundances), have been shown to actively degrade phosphonate substitutions from high molecular weight DOM, thereby generating an important source of P for the microbial community (Sosa et al., 2017). Increases in SAR116, associated with labile OM here (Figure 9H), has also been detected at appreciable abundances throughout the coastal and open gyre environments associated with the depths of maximum chlorophyll-a concentrations (Rappé et al., 1997; Morris et al., 2012; Nelson et al., 2014). Studies cited above demonstrate that responding taxa identified in the experiments here have also been shown to respond to a range of compounds within OM lending support to general decrease in various compound classes identified by LC-MS/MS here (Figures 3B, 7C).

### Increase in Community Diversity Favored During OM Degradation

Indicators of bacterioplankton diversity measured for dynamic marine environments can highlight the importance of the complex interactions associated with and between microbial networks spanning temporal and spatial scales (Chow et al., 2013; Fuhrman et al., 2015). We used the Shannon-Wiener *H* index as an indicator of diversity in the OM remineralization experiments to highlight the underlying importance of diversity associated with OM bioavailability. The diversity of the initial community increased significantly (*p* < 0.05; Willis, 2019) with depth in our experiments, with values (Table 3) similar to those reported for the Sargasso Sea (Nelson et al., 2014). An increasing bacterioplankton diversity over depth is a common feature in ocean systems and is likely influenced by a combination of slower speciation succession rates (Ghiglione et al., 2012) and elevated arrays of acquisition strategies as inorganic and organic resources change over depth (DeLong et al., 2006; Sogin et al., 2006).

**FIGURE 10 |:**
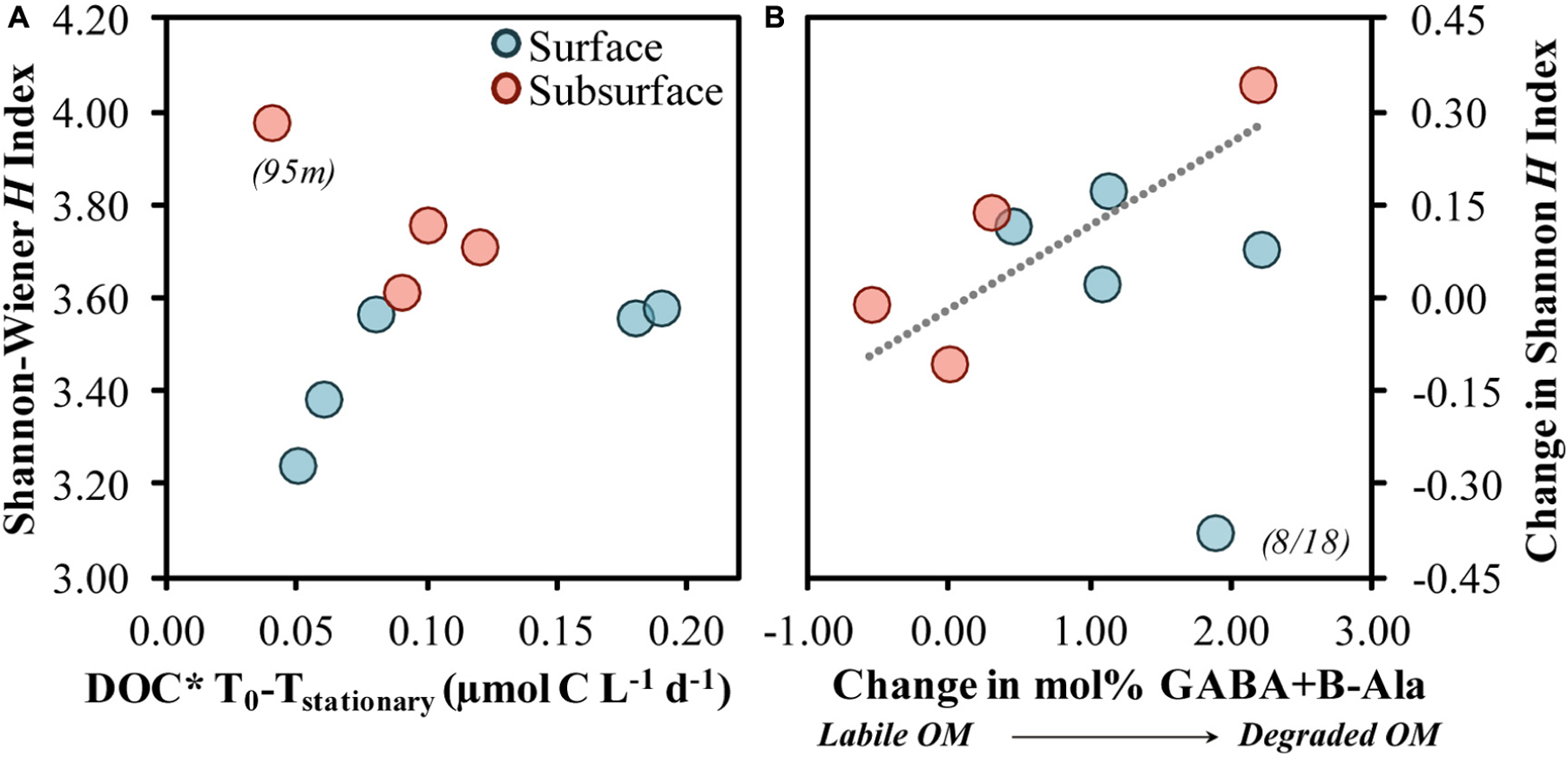
Shannon-Wiener *H* index at stationary phase associated with the short-term DOC* drawdown rate from diluted experiments **(A)** and the change in the Shannon-Wiener *H* index association with the change in the THAA-based mol% GABA + B-Ala **(B)**. The Model II linear fit line in **(B)** was significant (*p* = 0.029) when fit across both surface and subsurface samples but excludes the surface 8/18 sample. DOC* denotes that concentrations were corrected for bacterioplankton carbon but contain an unconstrained contribution of C < 3.0 μm.

The diversity of the responding taxa increased as DOC* drawdown rates increased from 0.05 to 0.10 μmol C L^−1^ d^−1^, after which point the diversity remained relatively constant to slightly decreasing (Figure 10A). The 95 m community sample contained elevated contributions of taxa (Figure 4) and OM (Figures 1, 2) not observed at other depths and so was not analyzed further as part of this trend. The pattern in Figure 10A is suggestive of the unimodal (“humped”) diversity-productivity relationship, where diversity increases to a point then stabilizes and decreases as the level of productivity increases (Smith, 2007). This pattern was recently illustrated for a range of bacterioplankton productivity conditions in arctic soils and was hypothesized to be due to shifts between stress tolerance with the lowest productivity and species competition at the highest productivity (Geyer and Barrett, 2019). The increase in diversity observed in the present study as DOC* drawdown began to increase may reflect an initial response to increasing bacterioplankton accessibility to OM resources. A shift to lower diversity at higher DOC* rates, dominated primarily by members within the *Gammaproteobacteria* class, has been shown in OM remineralization experiments associated with DOC removal rates 2–10 times greater than those reported here (Nelson et al., 2013; Wear et al., 2015).

Relative changes in diversity between initial and stationary growth phases were also greater as the changes in mol% GABA + B-Ala increased (Figure 10B). In other words, a greater alteration toward a more degraded OM was associated with an increasingly more diverse community. Increases in diversity as OM became more degraded may be reflective of an elevated number of specialists containing a diverse array of acquisition strategies (DeLong et al., 2006; Muscarella et al., 2019). The mole% increase by non-protein amino acids, GABA + B-Ala, implies a preferential modification of other protein-derived AAs, and may be in part due to the release of small compounds during the modification of protein acids like glutamic and aspartic acids (Lee and Cronin, 1982). Groups like OM43 have the ability to utilize simple organic acids (Gifford et al., 2016); their growth response in most experiments presented here may reflect either elevated organic acid concentrations in the NE Pacific (Koyama and Thompson, 1964) or a concurrent modification of OM by a diverse community that ultimately benefited OM43.

Diversity trends described above suggest that indicator species from a range of phylogenetic classes like *Bacteroidia* (e.g., *Flavobacteriaceae), Gammaproteobacteria* (e.g., *Alteromonas, OM43*) and *Alphaproteobacteria* (e.g., *Rhodobacteraceae*) can be favored in OM remineralization experiments (Figures 6, 9). Various acquisition strategies from a network of bacterioplankton are likely required to access the diverse molecular composition of OM (Fuhrman et al., 2015; Moran et al., 2016; Reintjes et al., 2019). For instance, ‘sharing’ bacteria like OM43 and *Alteromonadales Marinobacteraceae* use external hydrolysis to acquire necessary substrates, the process of which may benefit more ‘selfish’ taxa like *Flavobacteriaceae* (responding NS2b and NS4 genera identified here) that take up desired substrates with little of the substrate left unutilized during acquisition (Sosa et al., 2017; Reintjes et al., 2019; Arnosti et al., 2020; Liu and Liu, 2020). The dependence on diversity in the experiments shown here agrees with the idea that single species can take up substrate to a point but a diverse community is required to modify the complex composition of OM (Nelson and Wear, 2014; Pedler et al., 2014). Results from the current study suggest an elevated diversity was initially associated with increases in resource availability, but that this diversity would likely have decreased under a sustained supply of new OM (Wemheuer et al., 2014; Fuentes et al., 2019; Geyer and Barrett, 2019; Reji et al., 2020).

## CONCLUSION

Concurrent changes to the DOC* remineralization rate and to the THAA-based degradation index observed during the August 2018 EXPORTS study at OSP provide evidence that the rate of organic carbon removal by bacterioplankton was associated with the bioavailability/chemical composition of OM. Results agree with findings by Kirchman (1990), also based at OSP, demonstrating that amino acids, as opposed to sole additions of glucose, were a limiting component of OM to BP. We extend the findings from Kirchman (1990) by demonstrating that both the quantity and initial composition of the combined amino acid pool played an important role in determining the extent to which OM could be remineralized, where organic carbon removal rates were elevated when the initial combined amino acids indicated a more bioavailable composition.

We also extend observations of the bacterioplankton community at OSP by evaluating indicator 16S rDNA-based taxa associated with shifts in OM bioavailability. Similar to findings observed previously in both time-series and experimental studies associated with elevated resource availability, we found significant relationships between elevated OM bioavailability and members of the *Flavobacteriaceae* (NS2b genus), *Rhodobacteraceae* (*Sulfitobacter* genus), *Marinobacteraceae* (*Alteromonadales* order), *Methylophilaceae* (OM43 genus) and SAR116 families. By connecting the OM composition with the bacterioplankton community composition, it appears that a diverse set of taxa were responsible for changes to the OM composition.

These remineralization experiments also demonstrated that 16S amplicon-based community structures became more diverse as the OM exhibited the greatest changes in degradation state. Patterns of community diversity, with diverse resource acquisition strategies, may have contributed to relatively high BGE observed across all surface and subsurface experiments, averaging 31 ± 7%, similar to summer values measured previously for OSP (Sherry et al., 1999). Results, albeit from a limited data set, have implications on the BGEs and C cycling for the EXPORTS Program at the OSP study site, a site where bacterioplankton may at times contribute to net heterotrophic conditions (Fassbender et al., 2016) and BCD can exceed primary production rates (Sherry et al., 1999).

Further research on the bacterioplankton community at OSP would benefit from placing the carbon demand of the bacterioplankton into the broader carbon cycling context for region. For instance, bacterioplankton carbon removal and BGE estimates presented here will be used in a subsequent manuscript to constrain estimates bacterioplankton carbon demand and how that demand changes within the context of phytoplankton production rates, DOM concentration and DOM composition.

## Supplementary Material

Supplemental

## Figures and Tables

**TABLE 1 |:** Example sampling frequency schedule from the OM remineralization experiments.

	T0 to stationary growth phase		Stationary growth phase to 90 days	
	5 L Biotainer	40 mL parallel vial	5 L Biotainer	40 mL Parallel vial
DOC*	*N/A*	*Once per 2–3 days*	*N/A*	*Once per 14–30 days*
DAPI	*Once per 1 day*	*Once per 1 day*	*N/A*	*Once per 14–30 days*
FCM	*Once per 1 day*	*N/A*	*N/A*	*N/A*
THAA	*N/A*	*Once per 2–3 days*	*N/A*	*Once per 14–30 days*
DNA	*Initial/Stationary*	*N/A*	*N/A*	*N/A*
PPL	*Initial/Stationary*	*N/A*	*N/A*	*N/A*

Stationary phase occurred within 6–9 days of initiation and the final time point was around 90 days; the exact stationary and final time point days for each experiment are noted in Table 2. Bacterioplankton cell abundances were enumerated both by slide mounted DAPI-stained cells and flow cytometry (FCM).

**TABLE 2 |:** OM remineralization experiment variables: DOC*, DOC* removal rate, bacterioplankton biomass production rate and bacterioplankton growth efficiency.

Depth (m)	Start Date	Days to Stationary	Days to Final	Exp Type	DOC* (μmol C L^−1^)		DOC* Removal Rate (μmol C L^−1^ d^−1^)		Bacterioplankton Production Rate to Stationary (μmol C L^−1^ d^−1^)			Bacterioplankton Growth Efficiency (%)		
					T0	T-Final	T0 to Stationary	Stationary to Final	Gunder.	EXPORTS	Malfatti	Gunder.	EXPORTS	Malfatti
5	8/15	6	93	D	57.7 ± 0.4	56.6 ± 0.5	0.06 ± 0.04 n = 4, p = 0.21	–	0.09 ± 0.02	0.12 ± 0.02	0.16 ± 0.03	–	–	–
				U	60.4 ± 0.8	56.3 ± 0.2	0.14 ± 0.12 n = 4, p = 0.15	0.015 n = 5, p = 0.007	0.14 ± 0.01	0.19 ± 0.01	0.27 ± 0.02	–	–	–
	8/18	6	90	D	57.7 ± 0.4	56.0 ± 0.5	0.05 ± 0.07 n = 3, p = 0.54	0.014 n = 5, p = 0.03	0.05 ± 0.01	0.08 ± 0.02	0.12 ± 0.03	–	–	–
				U	60.0 ± 0.3	53.9 ± 0.4	0.38 ± 0.01 n = 3, p < 0.001	0.046 n = 4, p = 0.04	0.07 ± 0.01	0.10 ± 0.01	0.15 ± 0.01	0.18 ± 0.02	0.27 ± 0.03	0.39 ± 0.04
	8/23	10	85	D	57.8 ± 0.4	57.0 ± 0.5	0.08 ± 0.06 n = 3, p = 0.22	–	0.05 ± 0.01	0.07 ± 0.01	0.10 ± 0.01	–	–	–
				U	59.6 ± 0.4	57.1 ± 0.7	0.11 ± 0.02 n = 4, p = 0.005	0.021 n = 6, p = 0.009	0.03 ± 0.01	0.05 ± 0.01	0.07 ± 0.01	0.27 ± 0.09	0.45 ± 0.13	0.68 ± 0.18
	8/28	9	80	D	59.6 ± 0.5	56.8 ± 0.4	0.18 ± 0.03 n = 3, p = 0.003	0.016 n = 6, p = 0.03	0.03 ± 0.01	0.07 ± 0.01	0.11 ± 0.01	0.26 ± 0.06	0.38 ± 0.09	0.55 ± 0.13
				U	61.2 ± 0.4	56.8 ± 0.9	0.22 ± 0.06 n = 3, p = 0.008	0.031 n = 6, p = 0.01	0.04 ± 0.00	0.07 ± 0.01	0.11 ± 0.01	0.19 ± 0.05	0.32 ± 0.09	0.48 ± 0.13
	8/31	10	77	D	59.8 ± 0.6	55.0 ± 0.4	0.19 ± 0.05 n = 4, p = 0.003	0.044 n = 6, p < 0.001	0.03 ± 0.01	0.05 ± 0.01	0.07 ± 0.02	0.18 ± 0.07	0.27 ± 0.10	0.39 ± 0.14
				U	59.3 ± 0.4	54.3 ± 0.2	0.24 ± 0.06 n = 4, p = 0.003	0.039 n = 6, p = 0.01	0.04 ± 0.01	0.07 ± 0.01	0.10 ± 0.01	0.14 ± 0.05	0.27 ± 0.07	0.42 ± 0.11
35	8/23	10	85	D	58.0 ± 0.5	56.5 ± 0.6	0.09 ± 0.06 n **=** 3, p **=** 0.19	0.018 n **=** 4, p **=** 0.02	0.05 ± 0.01	0.06 ± 0.01	0.09 ± 0.01	–	–	–
				U	58.7 ± 0.5	55.9 ± 0.9	0.03 ± 0.02 n = 3, p = 0.29	0.017 n = 5, p = 0.08	0.02 ± 0.00	0.03 ± 0.00	0.05 ± 0.00	–	–	–
50	8/15	10	93	D	57.3 ± 0.4	56.3 ± 0.6	0.10 ± 0.02 n = 5, p = 0.003	0.014 n = 5, p = 0.18	0.03 ± 0.00	0.04 ± 0.01	0.05 ± 0.01	0.25 ± 0.06	0.35 ± 0.10	0.47 ± 0.12
				U	56.9 ± 0.5	54.7 ± 0.3	0.16 ± 0.03 n = 5, p = 0.003	0.021 n = 6, p = 0.05	0.02 ± 0.01	0.04 ± 0.01	0.06 ± 0.01	0.13 ± 0.05	0.25 ± 0.07	0.41 ± 0.11
	8/28	9	80	D	56.9 ± 0.4	54.0 ± 0.7	0.12 ± 0.09 n = 3, p = 0.25	0.023 n = 6, p < 0.001	0.01 ± 0.00	0.01 ± 0.01	0.01 ± 0.01	–	–	–
				U	58.6 ± 0.4	56.9 ± 0.3	0.20 ± 0.03 n = 3, p < 0.001	0.010 n = 6, p = 0.29	0.02 ± 0.00	0.02 ± 0.00	0.03 ± 0.00	0.25 ± 0.05	0.33 ± 0.06	0.46 ± 0.08
95	8/15	10	93	D	54.5 ± 0.3	54.3 ± 0.5	0.04 ± 0.03 n = 5, p = 0.49	–	0.01 ± 0.00	0.01 ± 0.01	0.01 ± 0.01	–	–	–
				U	54.2 ± 0.6	52.4 ± 0.6	0.13 ± 0.05 n = 5, p = 0.03	0.007 n = 5, p = 0.005	0.03 ± 0.00	0.04 ± 0.00	0.05 ± 0.00	0.13 ± 0.05	0.19 ± 0.08	0.27 ± 0.11

Bacterioplankton cell abundances and cell biovolumes were converted to cell C estimates either based on the relationship (“EXPORTS”) identified in Supplementary Figure 4 or based on published relationships (Gundersen et al., 2002) and (Malfatti et al., 2010). The range of bacterial growth efficiencies reflect estimates derived from various bacterioplankton carbon conversion factors determined empirically or from published values. BGE’s were only determined when the combined DOC* removal rates and bacterioplankton production rates were statistically significant (t-test p < 0.05).

D, diluted experiments; U, undiluted experiments.

DOC* denotes that concentrations were corrected for bacterioplankton carbon but contain an unconstrained contribution of C < 3.0 μm.

**TABLE 3 |:** Shannon-Wiener *H* Index, ±95% confidence intervals.

Depth	Date	Initial	Stationary	Significant Change
5 m	8/15/18	*3.36* ± *0.02*	*3.38* ± *0.02*	
	8/18/18	*3.62* ± *0.02*	*3.24* ± *0.01*	**
	8/23/18	*3.22* ± *0.02*	*3.57* ± *0.01*	**
	8/28/18	*3.42* ± *0.02*	*3.56* ± *0.02*	**
	8/31/18	*3.50* ± *0.01*	*3.58* ± *0.02*	**
35 m	8/23/18	*3.62* ± *0.01*	*3.61* ± *0.01*	
50 m	8/15/18	*3.58* ± *0.01*	*3.76* ± *0.02*	**
	8/28/18	*3.82* ± *0.01*	*3.71* ± *0.02*	**
95 m	8/15/18	*3.86* ± *0.02*	*3.98* ± *0.02*	**

** Initial and stationary phases were determined to be significantly different based on the Hutcheson’s t-tests at the p > 0.05 level.

## Data Availability

The data generated for this study can be found in the SeaWiFS Bio-optical and Storage System (SeaBASS, http://dx.doi.org/10.5067/SeaBASS/EXPORTS/DATA001). Profile and centroid LC-MS/MS is available through the massive repository (massive.ucsd.edu) under the following identifier: MSV000083365.
