# Supplementary material for: Organic Matter Composition at Ocean Station Papa Affects Its Bioavailability, Bacterioplankton Growth Efficiency and the Responding Taxa
Source: Front Mar Sci. Author manuscript; Available in PMC 2022 Jan 7. (PMC8740527; doi:10.3389/fmars.2020.590273)
Supplement: Supplemental [file NIHMS1763327-supplement-Supplemental.docx]

Supplementary Material

## Supplementary Text

**Sup. Text 1.** *LC-MS/MS additional methods description.*

LC-MS/MS data were acquired in positive mode with Top5 DDA MS/MS with MS1 resolution = 70000 and MS2 resolution = 17500. Non-targeted LC-MS/MS data were processed with the Ion-Identity Molecular Networking (IIMN) enabled version of MZmine2 (Schmid et al., 2020), and consensus MS/MS, IIMN edges and MS1 peak areas were submitted for Feature-based Molecular Networking through GNPS (Nothias et al., 2019). Initial intensity thresholds for feature extraction with MZmine were 1E5 for MS1 spectra and 1E3 for MS/MS spectra. MS1 chromatogram building was performed within 5 ppm mass windows and a minimum peak intensity was set to 3E5. Extracted Ion Chromatograms (XICs) were deconvoluted using the local minimum search algorithm with a chromatographic threshold of 1%, a search minimum in RT range of 0.1 min, minimum relative height of 10%, minimum absolute height of 3E5, minimum ratio of top/edge of 1.5 and a peak duration between 0.05 and 2 min.

For MS2 pairing, a median m/z center calculation with m/z range of 0.01 and retention time range was set to 0.1 min. Isotope peaks were grouped and features from different samples aligned with 5 ppm mass tolerance and 0.1 min retention time tolerance. MS1 peak lists were then aligned with an m/z tolerance of 5 ppm and retention time tolerance of 0.1 min with a weight of 75 on m/z and 25 on retention time. The feature list was then filtered to features with at least 2 isotope peaks and minimum occurrence in 2 at least LC-MS/MS files. Peak shape correlation of co-eluting features was performed with the metaCorrelate module; with a RT tolerance of 0.1, minimum height of 3E5 and noise level of 1E4. A correlation of 0.8 was set as the cut off for the min feature shape corr. The following adducts were considered for ion identities: [M + H+]+, [M + Na+]+, [M + K+]+ in combination with the neutral loss/additions of: [M-H2O] [M-CO], [M+ACN] and [M+FA]. Mass tolerance was set to 10 ppm, a maximum charge of 2, and maximum molecules/cluster of 3.

Peak areas and feature correlation pairs were exported as .csv files and the corresponding consensus MS/MS spectra as an .mgf file. Files were directly submitted for Ion-Identity Molecular Networking in GNPS (gnps.ucsd.edu). The precursor ion mass tolerance was set to 0.01 Da and the MS/MS fragment ion tolerance to 0.01 Da. A molecular network was then created where edges were filtered to have a cosine score above 0.70 and more than 6 matched peaks. Further, edges between two nodes were kept in the network if and only if each of the nodes appeared in each other’s respective top 10 most similar nodes. Finally, the maximum size of a molecular family was set to 100, and the lowest scoring edges were removed from molecular families until the molecular family size was below this threshold. The spectra in the network were then searched against GNPS spectral libraries and NIST17. The library spectra were filtered in the same manner as the input data. All matches kept between network spectra and library spectra were required to have a score above 0.7 and at least 5 matched peaks. The resulting molecular networking can be downloaded at: <https://gnps.ucsd.edu/ProteoSAFe/status.jsp?task=fc925fbe743343c9bba0059b90147956>.

## Supplementary Figures


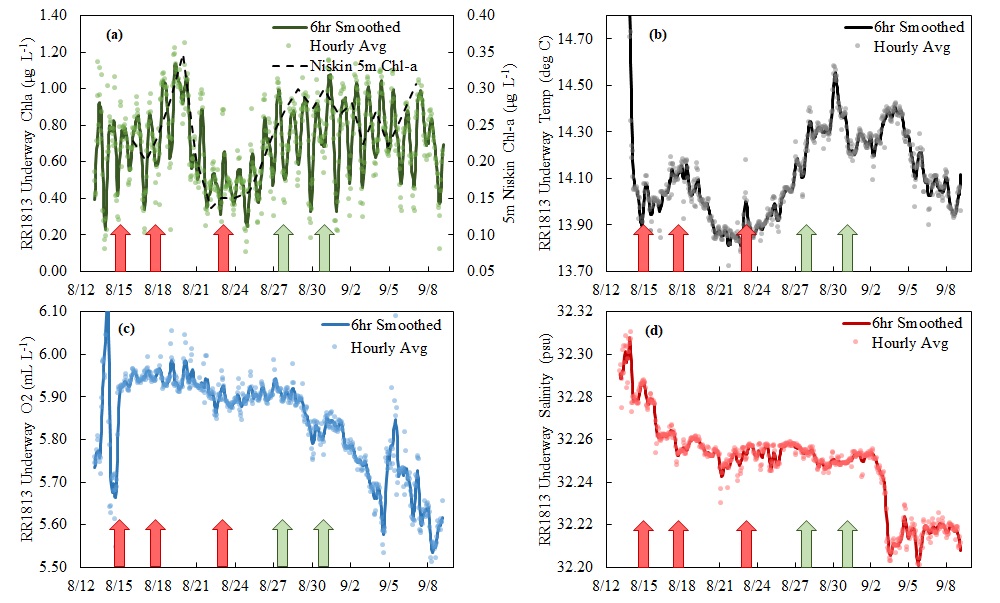


**Sup. Fig. 1.** *Surface flowthrough (~5 m) values of fluorescence (a), temperature (b), oxygen (c) and salinity (d). Arrows above x-axis denote when water was collected for DOM remineralization bioassays, where green arrows identify bioassays where a statistically significant DOC drawdown was observed over 0 d to stationary phase. Note that the 5m Niskin Chl-a data were collected from the afternoon (local) CTD cast, and it is likely that the underway Chl-a values should be adjusted down to match the Niskin Chl-a values.*


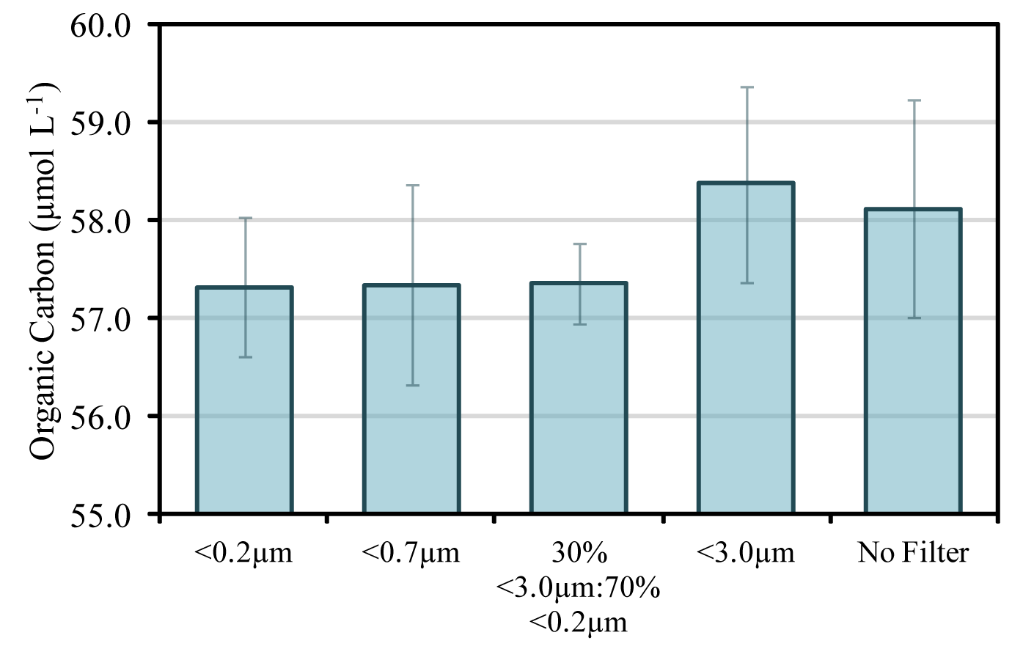


**Sup. Fig. 2.** *Initial C concentrations from an experiment initiated on Aug. 18, 2018. Values from the <0.2 µm and <3.0 µm filtrates were collected from 20 L carboys prior to mixing and the 30%:70% value reflects after mixing the two fractions. Values from the <0.7 µm and “No Filter” samples were collected directly from the Niskin bottle. Maximal differences between filtrates did not exceed 1.0 µmol C L^-1^.*


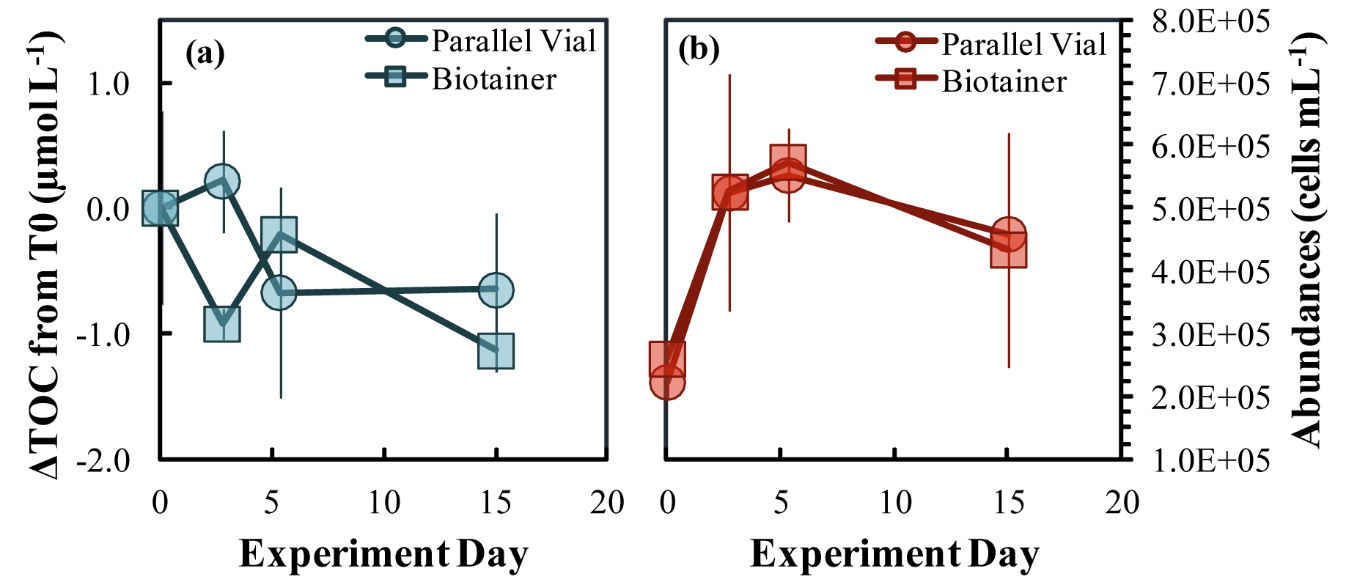


**Sup. Fig. 3.** *Changes in 5 m diluted bioassay [TOC] from the initial time point (T0) over ~16 days between 40 mL vials (i.e., borosilicate; “Parallel Vial”) and 5 L Nalgene (PC; “Biotainer”) bottles (a). Cell abundances were also collected from the Biotainer and parallel vials for the same time points (b). In most time-point-to-time-point comparisons the standard deviations on the values overlap [TOC] between Biotainer and the corresponding Parallel Vial. This experiment was initiated on Aug. 15, 2018.*


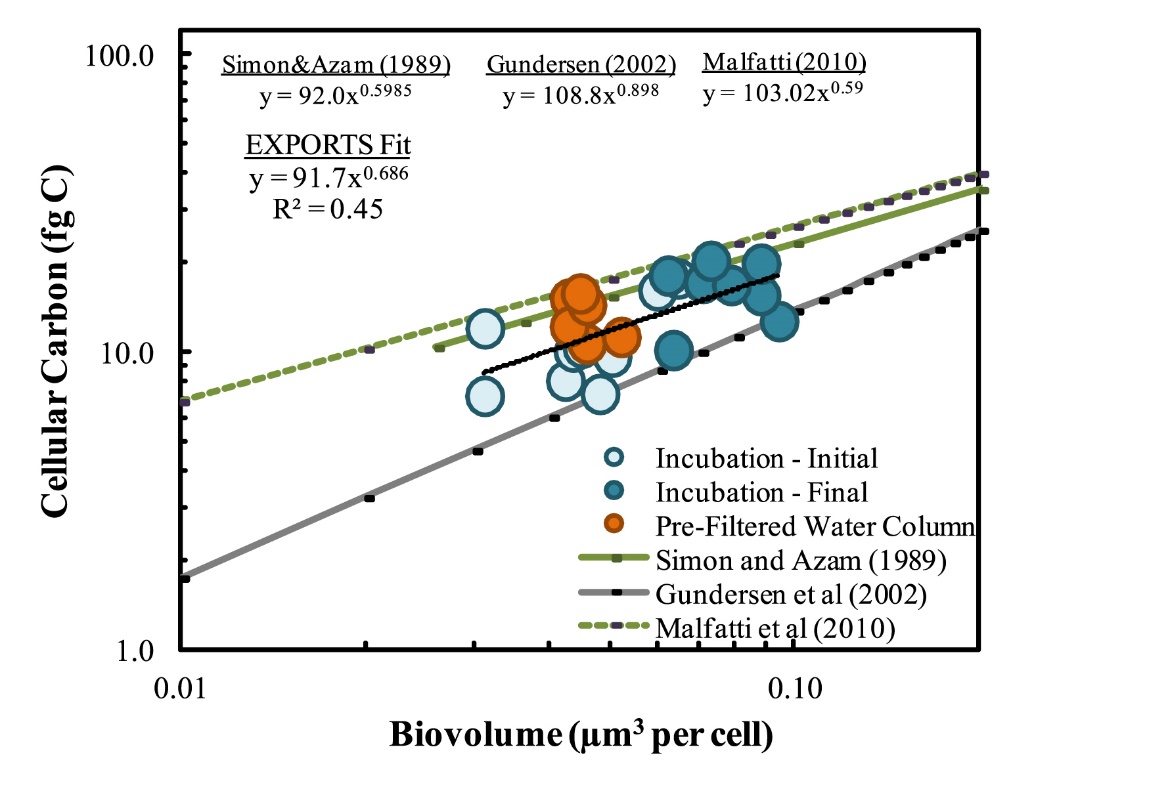


**Sup. Fig. 4.** *Cell biovolume determined from DAPI-stained images and calculated after Baldwin and Blankston (1988) plotted against cell carbon as determined concurrently from BC samples collected onto GF/75 filters (~0.3 µm nominal pore size). Also plotted are relationships determined previously by Simon and Azam (1989), Gundersen et al. (2002) and Malfatti et al. (2010). Axes are log scaled. Note that the EXPORTS fit falls between the relationships based on coastal communities collected near San Diego, CA (Simon & Azam and Malfatti) and oligotrophic communities collected from the Sargasso Sea (Gundersen). The Gundersen et al. (2002) and Malfatti et al. (2010) studies are based on DAPI-based stains, as was used in the current EXPORTS study, to estimate biovolume (compared with acridine orange stain in Simon and Azam (1989)) and so are included in Table 2.*


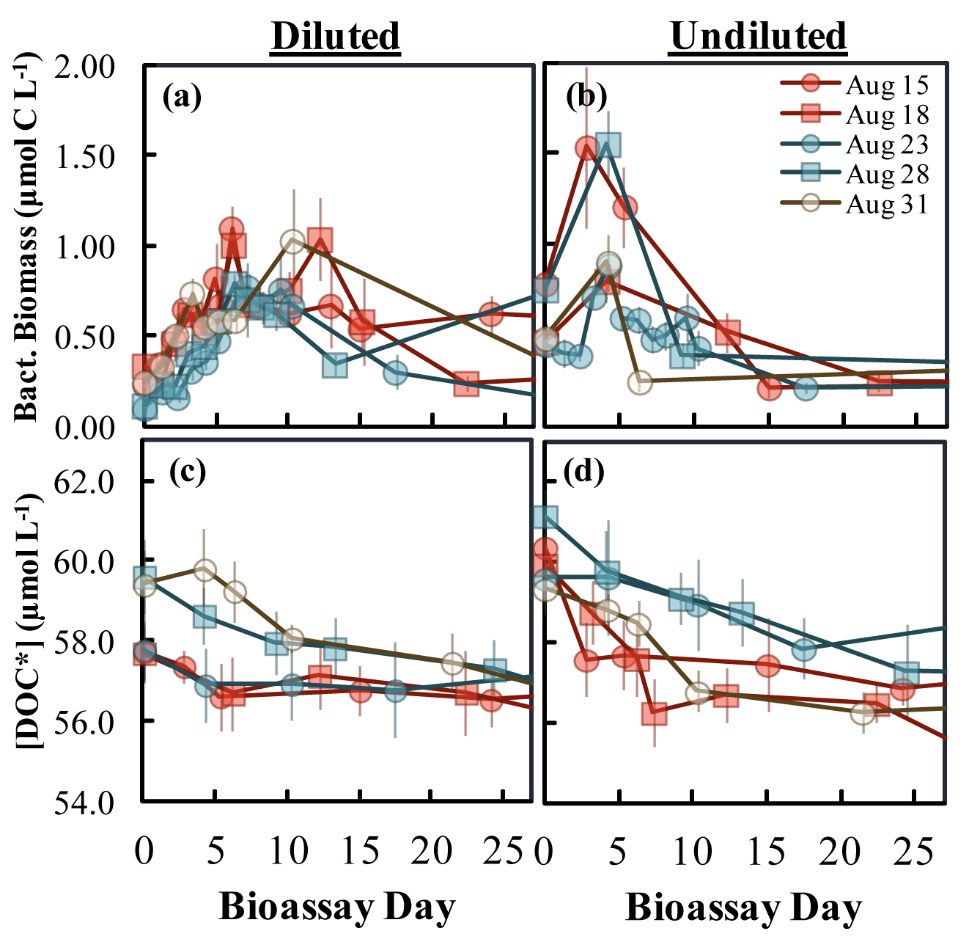


**Sup. Fig. 5.** *Temporal changes in mean bacterioplankton biomass (a, b) and [DOC*] (c, d) in surface OM remineralization experiments. “Diluted” bioassays (a, c) refer to incubations where 3.0 µm filtrate was diluted 70% with 0.2 µm filtrate and “undiluted” bioassays (b, d) refer to incubations of 3.0 µm filtrate only.* *DOC* denotes that concentrations were corrected for Bacterioplankton Biomass but contain an unconstrained contribution of C <3.0 µm.*

## Supplementary Tables

**Sup. Table 1.** *HPLC estimates of total hydrolyzable amino acids (THAA) as carbon, the degradation index (DI) score and mol% gamma-aminobutyric (GABA) and beta-alanine (B-Ala) from initial (0 d), stationary phase (6-10 d) and final time points (~90 d) from remineralization experiments. Experiment (Exp.) type refers to the diluted (‘D”; 30% 3.0 µm: 70% 0.2 µm) and undiluted (‘U’; 3.0 µm) experiments. The ‘*’ next to values indicates a significant (t-test, p<0.05) change to decreasing C and more degraded OM compared with 0 d and a ‘^’ indicates an increase in C and a change to more labile OM. Included errors represent the standard error values.*

| **Depth** | **Start Date** | **Exp. Type** | **THAA C (µmol L^-1^)** | | | **DI Score** | | | **Mol% GABA+B-Ala** | | |
| --- | --- | --- | --- | --- | --- | --- | --- | --- | --- | --- | --- |
| **(m)** |  |  | **0d** | **6-10d** | **~90d** | **0d** | **6-10d** | **~90d** | **0d** | **6-10d** | **~90d** |
| **5** | *8/15* | *D* | *1.1±0.5* | *1.0±0.1* | *0.7±0.1* | *2.3±0.2* | *1.5±0.1** | *0.4±0.2** | *5.2±0.2* | *6.3±0.1** | *8.9±0.4** |
|  | *8/18* | *D* | *1.1±0.4* | *1.0±0.1* | *0.6±0.1* | *1.7±0.1* | *0.8±0.3** | *-0.8±0.6** | *4.3±0.1* | *6.2±0.3** | *8.4±0.3** |
|  |  | *U* | *1.6±0.1* | *0.9±0.1** | *0.7±0.1** | *2.3±0.2* | *2.3±0.8* | *0.9±0.5** | *5.4±0.1* | *7.4±0.5** | *8.1±0.5** |
|  | *8/23* | *D* | *1.2±0.2* | *1.3±0.1* | *1.2±0.2* | *1.7±0.5* | *1.0±0.0* | *1.1±0.2* | *5.1±0.3* | *7.3±0.2** | *6.0±1.1* |
|  | *8/28* | *D* | *1.6±0.3* | *1.3±0.1* | *0.9±0.1** | *1.4±0.4* | *1.8±0.2* | *0.7±0.0** | *3.7±0.2* | *4.8±0.3** | *6.3±0.4** |
|  |  | *U* | *1.3±0.2* | *1.4±0.3* | *0.9±0.1** | *3.1±1.0* | *2.4±0.6* | *3.3±0.6* | *5.3±1.0* | *4.5±0.5* | *5.7±0.5* |
|  | *8/31* | *D* | *2.4±0.1* | *2.2±0.4* | *2.9±0.2^* | *3.4±0.4* | *3.9±0.9* | *4.4±0.2^* | *3.5±0.5* | *4.0±0.6* | *3.1±0.3* |
|  |  | *U* | *2.0±0.3* | *4.6±0.4^* | *1.0±0.3** | *2.0±0.4* | *2.6±0.4* | *2.2±0.4* | *3.6±0.0* | *1.4±0.1^* | *5.4±0.2** |
| **35** | *8/23* | *D* | *1.1±0.2* | *1.1±0.2* | *1.0±0.2* | *1.7±0.9* | *1.7±0.5* | *1.8±0.6* | *5.7±0.4* | *5.2±0.4* | *4.9±0.3* |
| **50** | *8/15* | *D* | *1.6±0.2* | *1.0±0.2* | *0.7±0.1** | *2.1±0.3* | *0.8±0.5** | *-0.3±0.5** | *4.2±0.2* | *5.4±0.4** | *7.0±0.4** |
| **95** | *8/15* | *D* | *0.9±0.2* | *0.8±0.0* | *1.0±0.1* | *-0.7±0.1* | *-0.4±0.4* | *-0.6±0.6* | *7.0±0.2* | *7.3±0.3* | *8.0±0.8* |

**Sup. Table 2.** *LC-MS/MS molecular features and associated compound classes based on InChI classifications of either library, analog or related molecular network features. Only those molecular features that had greater MS1 peak areas in surface samples are shown here.*

| Feature ID | m/z | Retention Time (min) | Zodiac Molecular Formula | Compound Class |
| --- | --- | --- | --- | --- |
| 2365 | 476.175688 | 8.499241 | C24H31NO5P2 | Amino |
| 2559 | 302.21165 | 4.529797 | C18H32P | Amino |
| 2837 | 186.11246 | 2.368862 | C9H15NO3 | Amino |
| 3794 | 224.164713 | 7.193678 | C13H21NO2 | Amino |
| 5396 | 416.316242 | 6.220628 | C26H41NO3 | Amino |
| 16733 | 400.248515 | 5.723333 | C24H33NO4 | Amino |
| 16742 | 242.138871 | 5.571198 | C12H19NO4 | Amino |
| 16751 | 490.316598 | 7.050062 | C28H43NO6 | Amino |
| 16760 | 240.159588 | 5.420339 | C13H21NO3 | Amino |
| 16778 | 218.13896 | 4.379001 | C10H19NO4 | Amino |
| 16784 | 158.117708 | 2.726429 | C8H15NO2 | Amino |
| 16785 | 227.104385 | 7.872671 | C13H16O2 | Amino |
| 16790 | 324.166179 | 4.456487 | C17H23N3O2 | Amino |
| 16807 | 409.064123 | 5.262835 | C15H21ClN2O5S2 | Amino |
| 16811 | 198.14891 | 5.525517 | C11H19NO2 | Amino |
| 16825 | 177.102462 | 2.035415 | C10H12N2O | Amino |
| 16831 | 198.148937 | 2.373932 | C11H19NO2 | Amino |
| 16855 | 368.226205 | 0.66093 | C16H33NO8 | Amino |
| 16857 | 450.285459 | 5.353626 | C21H35N7O4 | Amino |
| 16860 | 196.133269 | 4.436056 | C7H20N2O2P | Amino |
| 16863 | 174.055113 | 0.996917 | C5H13NO3 | Amino |
| 16908 | 245.113397 | 1.384697 | C10H16N2O5 | Amino |
| 16910 | 182.117672 | 5.023543 | C10H15NO2 | Amino |
| 16912 | 409.064029 | 5.412145 | C17H19N2O4P3 | Amino |
| 16929 | 184.133301 | 2.872139 | C10H17NO2 | Amino |
| 16950 | 152.107074 | 6.48485 | C9H13NO | Amino |
| 16951 | 464.337266 | 7.791886 | C23H41N7O3 | Amino |
| 16958 | 295.23828 | 3.687559 | C17H30N2O2 | Amino |
| 16972 | 191.117979 | 3.756946 | C11H14N2O | Amino |
| 17037 | 219.149437 | 3.72261 | C13H18N2O | Amino |
| 17041 | 161.107436 | 3.820623 | C10H12N2 | Amino |
| 17059 | 197.128523 | 3.484622 | C10H16N2O2 | Amino |
| 17075 | 196.065184 | 3.720352 | C9H11N2OS | Amino |
| 17106 | 161.107512 | 3.956248 | C10H12N2 | Amino |
| 17360 | 522.342785 | 6.962947 | C23H48N5O6P | Amino |
| 17406 | 185.08094 | 3.766281 | C9H12O4 | Amino |
| 17445 | 424.181086 | 8.25691 | C17H26N7O4P | Amino |
| 17869 | 282.119573 | 2.696553 | C11H19N2O5 | Amino |
| 17951 | 204.102061 | 6.25863 | C12H13NO2 | Amino |
| 18070 | 299.305906 | 7.337729 | C18H38N2O | Amino |
| 18484 | 174.091468 | 2.241967 | C11H11NO | Amino |
| 20441 | 217.09744 | 4.107371 | C12H12N2O2 | Amino |
| 29686 | 453.013593 | 5.562598 | C10H20N2O10P4 | Amino |
| 31453 | 275.160298 | 1.865393 | C12H22N2O5 | Amino |
| 31574 | 258.170271 | 5.040751 | C13H23NO4 | Amino |
| 41053 | 160.075832 | 1.381628 | C10H9NO | Amino |
| 47861 | 240.196016 | 4.429445 | C14H25NO2 | Amino |
| 64506 | 226.180366 | 3.428507 | C13H23NO2 | Amino |
| 85539 | 249.123519 | 2.972383 | C11H18N2O3 | Amino |
| 2234 | 205.122443 | 7.877228 | C13H16O2 | Aromatic |
| 6129 | 196.13327 | 6.036936 | C11H17NO2 | Aromatic |
| 10958 | 226.180359 | 3.426026 | C13H23NO2 | Aromatic |
| 16741 | 224.164698 | 8.016569 | C13H21NO2 | Aromatic |
| 16776 | 224.164702 | 8.228272 | C13H21NO2 | Aromatic |
| 16787 | 432.311106 | 5.291767 | C26H41NO4 | Aromatic |
| 16791 | 275.139181 | 7.11701 | C15H18N2O3 | Aromatic |
| 16792 | 203.11792 | 4.288691 | C12H14N2O | Aromatic |
| 16793 | 496.282333 | 5.626094 | C24H40N4O5P | Aromatic |
| 16803 | 196.133262 | 6.203579 | C11H17NO2 | Aromatic |
| 16866 | 180.138324 | 8.683865 | C11H17NO | Aromatic |
| 16869 | 165.102382 | 4.187974 | C9H12N2O | Aromatic |
| 16882 | 174.055051 | 2.85277 | C10H7NO2 | Aromatic |
| 16917 | 271.071742 | 2.629354 | C13H15NO2P | Aromatic |
| 16984 | 248.077965 | 0.897928 | C9H13NO7 | Aromatic |
| 16998 | 236.147827 | 3.654376 | C10H21NO5 | Aromatic |
| 17021 | 242.175278 | 2.239529 | C13H23NO3 | Aromatic |
| 17046 | 293.068872 | 8.467253 | C12H18ClO4P | Aromatic |
| 17133 | 204.07698 | 4.259714 | C10H9N3O2 | Aromatic |
| 17203 | 219.076676 | 4.233518 | C9H15O4P | Aromatic |
| 17214 | 178.086346 | 4.00268 | C10H11NO2 | Aromatic |
| 17232 | 474.22525 | 7.94447 | C24H31N3O7 | Aromatic |
| 17314 | 179.081654 | 1.013569 | C9H10N2O2 | Aromatic |
| 17735 | 275.139196 | 7.186752 | C15H18N2O3 | Aromatic |
| 20447 | 259.128945 | 2.829788 | C11H18N2O5 | Aromatic |
| 75471 | 235.144315 | 3.636485 | C13H18N2O2 | Aromatic |
| 3842 | 376.248629 | 5.706511 | C22H33NO4 | Lipid |
| 4023 | 193.133659 | 2.547115 | C11H16N2O | Lipid |
| 4215 | 241.143598 | 8.363397 | C9H23NO4P | Lipid |
| 5166 | 228.159702 | 1.024703 | C12H21NO3 | Lipid |
| 5717 | 362.232909 | 4.855885 | C21H31NO4 | Lipid |
| 9279 | 535.173947 | 8.692548 | C21H28N8O5P2 | Lipid |
| 16749 | 388.284795 | 5.468587 | C24H37NO3 | Lipid |
| 16763 | 184.096909 | 4.026612 | C9H13NO3 | Lipid |
| 16765 | 376.284958 | 5.041422 | C23H37NO3 | Lipid |
| 16797 | 205.097282 | 5.448945 | C11H12N2O2 | Lipid |
| 16804 | 228.159863 | 0.671146 | C12H21NO3 | Lipid |
| 16830 | 365.207327 | 4.792545 | C19H28N2O5 | Lipid |
| 16849 | 325.237461 | 8.520902 | C19H32O4 | Lipid |
| 16899 | 229.121448 | 7.201153 | C9H17N4OP | Lipid |
| 16920 | 512.340414 | 6.280471 | C19H46N9O5P | Lipid |
| 16924 | 241.126074 | 6.143423 | C7H18N3O6 | Lipid |
| 16940 | 402.300442 | 7.846647 | C25H39NO3 | Lipid |
| 16961 | 275.185422 | 5.746359 | C14H26O5 | Lipid |
| 16967 | 203.081522 | 5.80685 | C11H10N2O2 | Lipid |
| 17023 | 398.305575 | 9.539091 | C26H39NO2 | Lipid |
| 17241 | 402.30042 | 8.943312 | C25H39NO3 | Lipid |
| 17395 | 402.300449 | 8.166719 | C12H37N10O5 | Lipid |
| 17539 | 402.239033 | 2.209018 | C22H31N3O4 | Lipid |
| 18027 | 451.324179 | 8.96264 | C27H48OP2 | Lipid |
| 18069 | 558.323729 | 8.972064 | C26H50N5O2P3 | Lipid |
| 19120 | 198.112565 | 5.050828 | C10H15NO3 | Lipid |
| 20442 | 285.290251 | 6.682122 | C17H36N2O | Lipid |
| 32839 | 228.159861 | 0.671146 | C12H21NO3 | Lipid |
| 42268 | 218.092739 | 5.996332 | C11H11N3O2 | Lipid |
| 80714 | 299.167724 | 3.731064 | C16H26O3S | Lipid |
| 16781 | 191.081542 | 3.625476 | C10H10N2O2 | Peptide |
| 16835 | 246.146671 | 8.017817 | C13H21NO2 | Peptide |
| 16841 | 191.081547 | 4.644486 | C10H10N2O2 | Peptide |
| 17031 | 246.146671 | 8.228301 | C13H21NO2 | Peptide |
| 17216 | 243.098527 | 0.926034 | C10H14N2O5 | Peptide |
| 17542 | 247.129115 | 0.901361 | C10H18N2O5 | Peptide |
| 32930 | 406.214295 | 5.362496 | C22H32NO4P | Peptide |
| 79952 | 191.081538 | 3.625476 | C10H10N2O2 | Peptide |
| 11145 | 400.24832 | 5.903681 | C24H33NO4 | Steroid |
| 17381 | 438.285118 | 8.931171 | C24H39NO6 | Steroid |
| 76227 | 408.229222 | 6.25035 | C16H35N5O3P2 | Steroid |
| 16801 | 339.253116 | 9.35785 | C20H34O4 | Terpenoid |
| 16823 | 308.25861 | 7.015882 | C19H33NO2 | Terpenoid |
| 16960 | 268.190916 | 5.413072 | C15H25NO3 | Terpenoid |
| 17009 | 375.043154 | 8.443924 | C16H19O3P3 | Terpenoid |
| 17114 | 281.174854 | 4.959645 | C16H24O4 | Terpenoid |
| 17233 | 335.050478 | 7.720641 | C12H20NO2P4 | Terpenoid |
| 17239 | 303.231925 | 9.351182 | C20H30O2 | Terpenoid |
| 18041 | 289.216261 | 8.529311 | C19H28O2 | Terpenoid |
| 32675 | 240.159584 | 6.373554 | C13H21NO3 | Terpenoid |
| 14865 | 506.214797 | 7.214864 | C24H31N3O9 | Unknown |
| 16737 | 455.011103 | 5.425858 | C14H18ClN2O7P3 | Unknown |
| 16743 | 414.979946 | 4.509055 | C12H14O10S3 | Unknown |
| 16745 | 513.33255 | 5.869143 | C30H44N2O5 | Unknown |
| 16747 | 367.161238 | 4.317937 | C18H24NO7 | Unknown |
| 16753 | 456.274775 | 5.965118 | C22H43NO5S | Unknown |
| 16769 | 455.011162 | 5.582638 | C11H14N6O8S3 | Unknown |
| 16772 | 558.207449 | 6.286686 | C29H38NO4P3 | Unknown |
| 16775 | 433.269949 | 5.48399 | C24H36N2O5 | Unknown |
| 16786 | 442.258925 | 4.613039 | C24H33N4O4 | Unknown |
| 16813 | 369.032874 | 4.296096 | C14H15N2O4P3 | Unknown |
| 16875 | 268.030763 | 4.319538 | C7H9NO10 | Unknown |
| 16888 | 461.100763 | 7.487307 | C23H17N4O5P | Unknown |
| 16890 | 291.155202 | 1.157087 | C12H22N2O6 | Unknown |
| 16891 | 335.050447 | 8.447359 | C12H20NO2P4 | Unknown |
| 16906 | 414.204365 | 10.20847 | C22H27N3O5 | Unknown |
| 16911 | 554.351293 | 7.791523 | C21H41N14O4 | Unknown |
| 16919 | 227.079268 | 5.45249 | C9H17O3P | Unknown |
| 16946 | 328.227384 | 7.668486 | C21H29NO2 | Unknown |
| 16947 | 349.212384 | 5.301343 | C19H28N2O4 | Unknown |
| 17001 | 402.300507 | 7.479536 | C25H39NO3 | Unknown |
| 17028 | 391.230901 | 5.948923 | C19H34O8 | Unknown |
| 17054 | 558.207275 | 6.047511 | C24H44NO5P3 | Unknown |
| 17115 | 263.139126 | 5.970833 | C7H24N5P2 | Unknown |
| 17127 | 386.269074 | 7.999579 | C24H35NO3 | Unknown |
| 17129 | 335.07178 | 2.9527 | C12H23ClO3P2 | Unknown |
| 17156 | 296.13546 | 0.66691 | C14H19N2O5 | Unknown |
| 17173 | 766.175355 | 8.401105 |  | Unknown |
| 17180 | 190.107473 | 1.641244 | C8H15NO4 | Unknown |
| 17185 | 414.077428 | 9.124405 | C13H20NO12P | Unknown |
| 17265 | 522.20976 | 6.8376 | C22H36NO11P | Unknown |
| 17273 | 792.453124 | 6.748178 | Unknown | Unknown |
| 17279 | 421.218493 | 1.139396 | C18H32N2O9 | Unknown |
| 17333 | 547.154825 | 10.06885 | C20H23N10O7P | Unknown |
| 17374 | 528.265482 | 0.962904 | C18H37N7O11 | Unknown |
| 17421 | 368.225904 | 1.393142 | C20H35NOP2 | Unknown |
| 17441 | 278.123101 | 2.495928 | C4H14N12OP | Unknown |
| 17622 | 478.217558 | 9.29886 | C23H31N3O8 | Unknown |
| 17698 | 372.039473 | 3.08891 | C14H15NO7P2 | Unknown |
| 17937 | 494.329874 | 7.872049 | C23H45N5O5 | Unknown |
| 18016 | 402.300539 | 7.568719 | C12H37N10O5 | Unknown |
| 18028 | 494.32996 | 6.953996 | C24H48NO7P | Unknown |
| 18311 | 254.113811 | 2.092431 | C11H15N3O4 | Unknown |
| 18402 | 260.105412 | 4.838255 | C11H18NO4P | Unknown |
| 19085 | 448.282611 | 6.207612 | C24H37N3O5 | Unknown |
| 19763 | 445.241323 | 3.672676 | C17H37N5O5P | Unknown |
| 19877 | 520.082835 | 8.863429 | C23H21O7P3 | Unknown |
| 20109 | 362.269249 | 7.247816 | C22H35NO3 | Unknown |
| 33133 | 402.30047 | 7.418348 | C10H35N13O4 | Unknown |
| 36887 | 362.232769 | 4.058051 | C6H27N13O5 | Unknown |
| 41111 | 402.189265 | 9.158395 | C19H30N3O3P | Unknown |
| 41604 | 474.225048 | 7.918389 | C24H31N3O7 | Unknown |
| 41620 | 315.191636 | 3.253922 | C15H26N2O5 | Unknown |
| 43514 | 448.282704 | 6.22688 | C24H37N3O5 | Unknown |
| 44934 | 366.227882 | 4.407437 | C20H31NO5 | Unknown |
| 44994 | 255.08004 | 1.952772 | C11H16N2OP2 | Unknown |
| 45572 | 367.088386 | 3.889597 | C15H24NO3P2 | Unknown |
| 53598 | 232.096968 | 2.844729 | C11H17N2O | Unknown |
| 57527 | 525.038197 | 8.861363 | C19H24NO7P4 | Unknown |
| 57541 | 521.066911 | 6.421821 | C23H23O8P3 | Unknown |
| 58268 | 503.0562 | 8.859873 | C23H21O7P3 | Unknown |
| 84678 | 378.227039 | 10.25241 | C19H37O3S2 | Unknown |
| 84731 | 752.213787 | 8.144615 |  | Unknown |
| 113830 | 430.199173 | 8.613285 | C22H27N3O6 | Unknown |
